# Remodeling of the Enterococcal Cell Envelope during Surface Penetration Promotes Intrinsic Resistance to Stress

**DOI:** 10.1128/mbio.02294-22

**Published:** 2022-11-10

**Authors:** Yusibeska Ramos, Stephanie Sansone, Sung-Min Hwang, Tito A. Sandoval, Mengmeng Zhu, Guoan Zhang, Juan R. Cubillos-Ruiz, Diana K. Morales

**Affiliations:** a Department of Obstetrics and Gynecology, Weill Cornell Medicine, New York, New York, USA; b Department of Urology, Weill Cornell Medicine, New York, New York, USA; c Proteomics and Metabolomics Core Facility, Weill Cornell Medicine, New York, New York, USA; d Sandra and Edward Meyer Cancer Center, Weill Cornell Medicine, New York, New York, USA; e Immunology and Microbial Pathogenesis Program, Weill Cornell Graduate School of Medical Sciences, Weill Cornell Medicine, New York, New York, USA; University of Texas Health Science Center at Houston

**Keywords:** aggregates, cell envelope, daptomycin, defensins, *Enterococcus*, glycerophospholipids, glycolipids, membrane lipids, surface-penetration, translocation, ultrastructural

## Abstract

Enterococcus faecalis is a normal commensal of the human gastrointestinal tract (GIT). However, upon disruption of gut homeostasis, this nonmotile bacterium can egress from its natural niche and spread to distal organs. While this translocation process can lead to life-threatening systemic infections, the underlying mechanisms remain largely unexplored. Our prior work showed that E. faecalis migration across diverse surfaces requires the formation of matrix-covered multicellular aggregates and the synthesis of exopolysaccharides, but how enterococcal cells are reprogrammed during this process is unknown. Whether surface penetration endows E. faecalis with adaptive advantages is also uncertain. Here, we report that surface penetration promotes the generation of a metabolically and phenotypically distinct E. faecalis population with an enhanced capacity to endure various forms of extracellular stress. Surface-invading enterococci demonstrated major ultrastructural alterations in their cell envelope characterized by increased membrane glycolipid content. These changes were accompanied by marked induction of specific transcriptional programs enhancing cell envelope biogenesis and glycolipid metabolism. Notably, the surface-invading population demonstrated superior tolerance to membrane-damaging antimicrobials, including daptomycin and β-defensins produced by epithelial cells. Genetic mutations impairing glycolipid biosynthesis sensitized E. faecalis to envelope stressors and reduced the ability of this bacterium to penetrate semisolid surfaces and translocate through human intestinal epithelial cell monolayers. Our study reveals that surface penetration induces distinct transcriptional, metabolic, and ultrastructural changes that equip E. faecalis with enhanced capacity to resist external stressors and thrive in its surrounding environment.

## INTRODUCTION

Genetically identical individuals within bacterial populations exhibit phenotypic heterogeneity that provides a dynamic source of diversity (1, 2). This variability can engender subpopulations that are better equipped to cope with unpredictable conditions and/or exploit new environments (3). Phenotypic variation among pathogens dictates their virulence, their resistance to antimicrobials and other stressors, and their capability to express motility determinants (1, 2, 4, 5). Enterococcus faecalis, for instance, is a highly adaptable bacterial pathobiont that can endure an extensive range of adverse conditions (6–8). This bacterium is found in the gastrointestinal tract (GIT), as well as in the oral cavity and urinary and vaginal tracts of multiple organisms (6, 8). In the GIT of humans and mice, enterococci primarily colonize the small and large intestine (9, 10), which could be mediated by the formation of biofilm-like aggregates (9). As an intestinal commensal, this bacterium faces extreme challenges, including the constant turnover of GIT content, exposure to antimicrobial peptides produced by the host and bacterial neighbors, and intense competition for nutrients by other intestinal coinhabitants. E. faecalis has a remarkable ability to tune itself to overcome gut challenges and persist in this changing milieu (11).

E. faecalis can overgrow in the GIT due to immunosuppression or during prolonged antibiotic treatment. These conditions can perturb mucosal immunity and integrity and alter the normal commensal microbiota, and thus facilitate intestinal domination by drug-resistant enterococcal strains that can breach the intestinal epithelial barrier (12–14). This specialized process of intestinal egress, called translocation, enables E. faecalis to access the bloodstream and colonize distal anatomical sites (15–17), causing life-threatening diseases such as bacteremia and nosocomial infections (18, 19). Notably, translocation is a multifactorial process involving host pathways and intrinsic bacterial mechanisms that contribute to persistence and escape from the intestinal epithelial barrier (13, 17, 20).

Our group developed various *ex vivo* model systems that allow a detailed mechanistic analysis of the molecular events that render nonmotile E. faecalis capable of penetrating semisolid surfaces and translocating through intestinal epithelial barriers (21). We established that this penetration process is accompanied by the assembly of specialized, three-dimensional multicellular aggregates covered by a self-produced matrix partly composed of exopolymers, which are necessary for optimal enterococcal migration (21). These aggregates, comparable to the structures previously described during enterococcal GIT colonization in gnotobiotic mice (9), assembled inside the semisolid agar surface and during translocation across epithelial barriers, localizing throughout surface invaginations and openings within the epithelial monolayers (21). Notably, our analyses using a two-chamber transcytosis system (21, 22) demonstrated that only a fraction of the total enterococcal cells were capable of passing through intact intestinal epithelial barriers *in vitro* (21). Similarly, on semisolid agar surfaces, whereas few enterococci remained outside and formed regular colonies on the medium, another group readily penetrated inside the semisolid surface (21). Nonetheless, how enterococci are physiologically programmed during this process is unknown. Whether surface penetration endows E. faecalis with adaptive advantages also remains elusive.

Here, we report that surface penetration promotes the generation of a phenotypically distinct enterococcal population with enhanced capacity to endure various forms of extracellular stress. Surface-penetrating enterococci differed from their external counterparts in their cell envelope (CE) ultrastructure and lipid composition. We identified unique gene programs and lipid profiles in the penetrating community that increased glycolipid abundance and conferred superior tolerance to membrane-damaging agents. Importantly, an E. faecalis mutant unable to produce glycolipids demonstrated reduced antimicrobial resistance as well as impaired ability to translocate through human epithelial cell monolayers.

## RESULTS

### Surface-penetrating enterococci exhibit ultrastructural changes in their CE.

**FIG 1:**
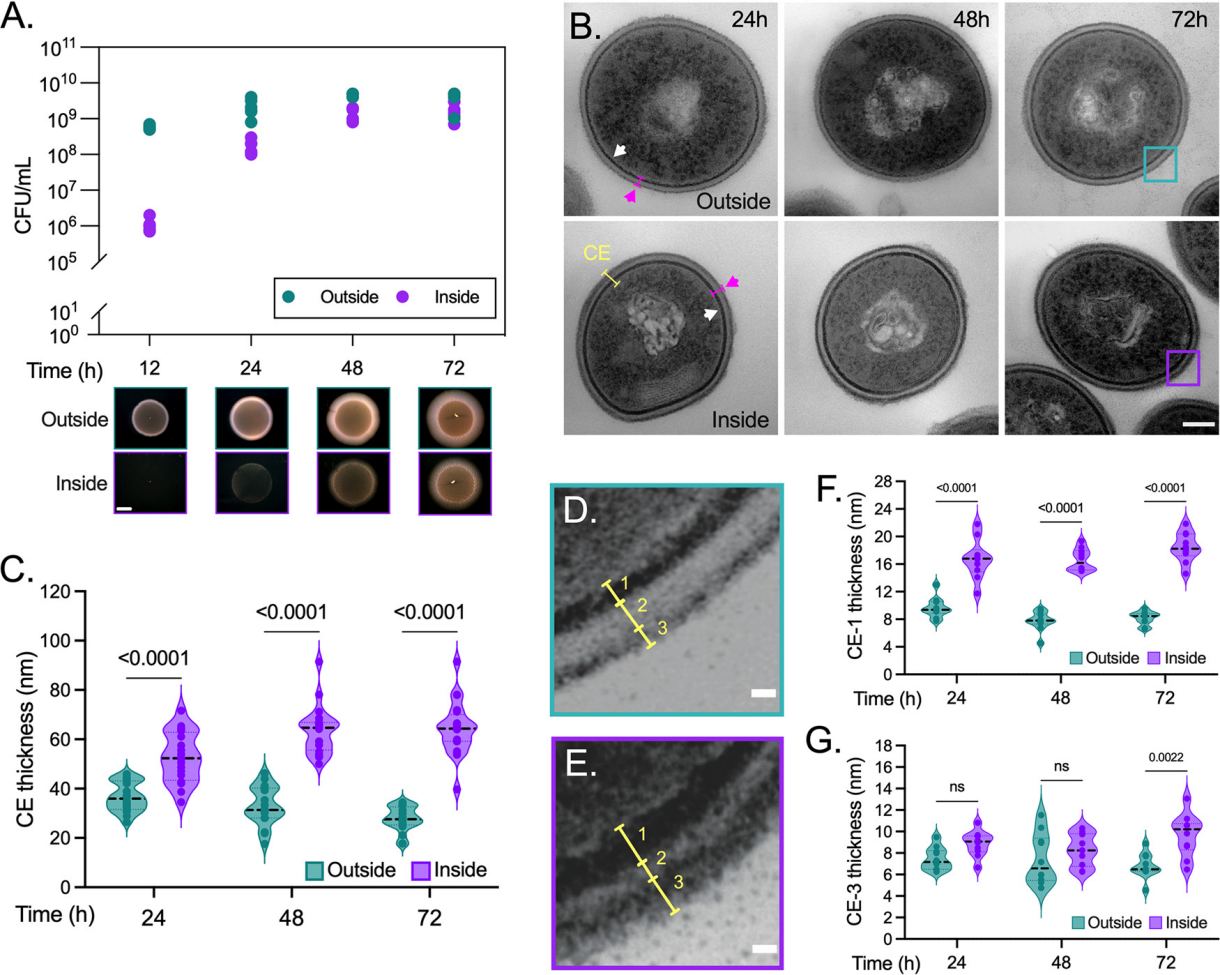
Semisolid surface penetration prompts cell envelope (CE) ultrastructural alterations in E. faecalis. (A) The enterococcal capacity to penetrate MOLP, evidenced as a colony-print inside the semisolid medium (bottom, lower panel) after removing the nonpenetrating bacteria (outside; bottom, upper panel), was quantified over time and expressed as CFU/mL (top; *n* = 3). (B) Electron micrographs of enterococcal cells from the surface and inside MOLP recovered at the indicated time postinoculation. Arrows show the localization of the plasma membrane (white) and cell wall (magenta), two components of the bacterial CE (yellow brackets). (C) CE thickness differences between agar-penetrating and nonpenetrating enterococci (*n* = 20). (D and E) Cyan and purple squares indicate the magnified areas of TEM images taken at 72 h from panel (B) of invading (E, *bottom*) and noninvading (D, top) enterococci. A three-zone CE (yellow bracket) characterized by an inner electron-dark zone (1), followed by an intermediate electron-translucent area (2) and an outermost fibrous heavily stained layer (3) is highlighted in each case. (F and G) Measurements of the width of CE electron-dark zones 1 and 3 over time, expressed in nm (*n* = 10). (C, F and G) Two-way analysis of variance (ANOVA; Tukey’s test); ns, nonsignificant. Each data point represents an independent biological replicate. Scale bars = (A) 2000 μm, (B) 100 nm, and (D and E) 10 nm.

We reported that nonmotile enterococcal isolates, including the commensal-like strain E. faecalis OG1RF, readily penetrate and invade semisolid surfaces, which was evidenced as an indelible bacterial “colony-print” (Fig. 1A, inside; 21). Even after 6 days of incubation, some bacteria remained on top of the semisolid agar and developed regular colonies (Fig. 1A, outside; 21), suggesting that a distinct enterococcal population might undergo early phenotypic variation that enables effective surface penetration. We sought to test this underappreciated possibility. Using E. faecalis OG1RF as a model system, we quantified the colony forming units (CFU) from bacteria located inside or outside semisolid medium optimal for lipopeptide production (MOLP) at earlier time points after inoculation. Although the colony-print was not macroscopically evident (Fig. 1A, bottom), E. faecalis could be recovered from inside the agar as early as 12 h after inoculation. (Fig. 1A, top). The CFU obtained for this population (~1 × 10^6^) were reproducibly 2-fold lower than those of the external cells (~6 × 10^8^; Fig. 1A). However, the number of surface-penetrating cells increased over time to finally reach numbers similar to those of enterococci remaining outside the agar surface at 72 h (Fig. 1A, top). Transmission electron microscopy (TEM) showed that the total area of the cells penetrating the agar for 24 h was significantly lower than that of the cells growing on the surface, but these size differences were no longer evident at later time points (Fig. 1B and Fig. S1A in the supplemental material). Importantly, we found that surface-penetrating bacteria demonstrated major ultrastructural alterations in their periphery: cells growing inside the semisolid surface exhibited a thicker CE at all time points evaluated than their nonpenetrating counterparts remaining on the agar surface (Fig. 1B and C). As described in other Gram-positive bacteria (23, 24), a three-zoned CE characterized by an inner electron-dark zone (CE-1; Fig. 1D and E), followed by an intermediate electron-translucent area (CE-2; Fig. 1D and E), and an outermost fibrous heavily stained layer (CE-3; Fig. 1D and E) was observed at high magnifications. Whereas CE-2 remained unaltered over time (Fig. S1B), the length of CE-1 and CE-3 significantly increased (~2.5- and ~1.5-fold, respectively) in surface-penetrating cells, compared with their external counterparts, 72 h after inoculation (Fig. 1F and G). CE-1 was consistently thicker in enterococci extracted from the colony-print than in the external bacteria at 24 and 48 h (Fig. 1F). Hence, surface penetration by E. faecalis subpopulations is accompanied by major CE ultrastructural alterations.

10.1128/mbio.02294-22.1 FIG S1 E. faecalis cell envelope (CE) ultrastructural changes over time. (A and B) TEM images from external (outside) and agar-penetrating (inside) enterococcal cells were analyzed to determine the total area of their transversal sections (A) and the thickness of the CE electron-translucent zone 2 (B; CE-2, depicted in Fig. 1). Cell area was calculated using the formula area = *ab* Π, where *a* and *b* denote the major and minor radiuses of the ellipsoidal cell, respectively (a > b) (A; right diagram). Two-way ANOVA (Tukey’s test) was used for all data; *n* = 10 and *n* = 20 for panels A and B, respectively. ns, not significant. Each data point represents an independent biological replicate. Download FIG S1, TIF file, 0.2 MB. Copyright © 2022 Ramos et al. 2022 Ramos et al. https://creativecommons.org/licenses/by/4.0/ This content is distributed under the terms of the Creative Commons Attribution 4.0 International license.

### Surface penetration induces discrete CE modifications in E. faecalis.

**FIG 2:**
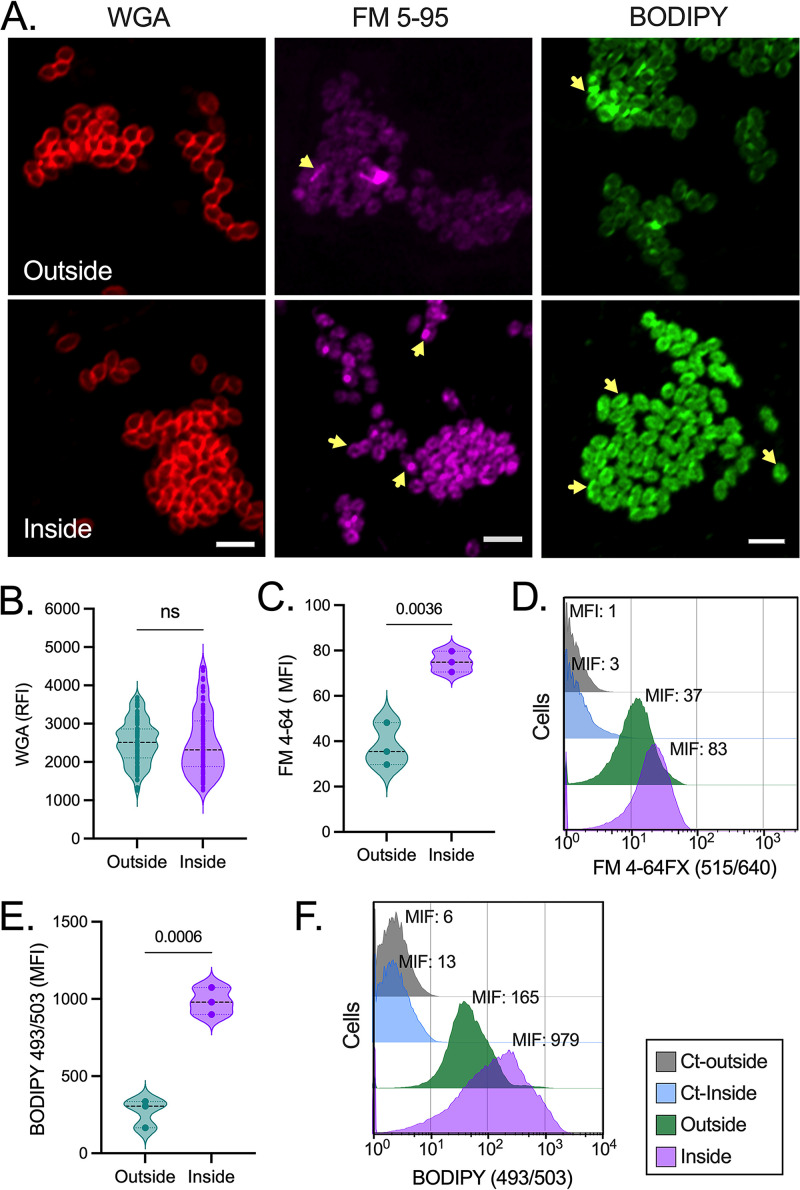
Alterations in E. faecalis neutral and anionic membrane lipids during surface penetration. (A to F) Bacteria were recovered from inside the agar or the surface (outside) 72 h after inoculation on MOLP, and cells were stained with either Texas Red-conjugated WGA (A, left; B), FM 5-95 (A, center; C and D), or BODIPY 493/503 (A, right; E and F). (A) Confocal fluorescence microscopy analyses of agar-penetrating (inside, bottom) and nonpenetrating (outside, top) E. faecalis. Yellow arrows indicate areas of robust staining. Scale bar = 2.0 μm. (B) The relative fluorescence intensity (RFI) of WGA-stained single cells from CLSM images of agar-invading and external enterococci was measured using Fiji (*n* = 100; two-tailed unpaired *t* test). The corresponding fluorescence of cells stained with either anionic (C and D) or neutral (E and F) lipid-specific dyes was quantified using flow cytometry (C and E) and expressed as mean fluorescence intensity (MFI; D and F). Each data point represents an independent biological replicate (*n* = 3); two-tailed unpaired *t* test; ns, not significant. Data are representative of at least three independent experiments.

The bacterial CE is a complex, multilayered, and dynamic structure encompassing the cell wall and plasma membrane (25). We examined differences in this structure by microscopically analyzing the membrane and cell wall components of external versus surface-penetrating E. faecalis cells. First, we used lectin wheat germ agglutinin (WGA), which binds to bacterial cells (26–28) in part by interacting with the *N-*acetylglucosamine-containing polymers that constitute the peptidoglycan layer of the cell wall (29). Confocal laser scanning microscopy (CLSM) analyses revealed a similar distribution of WGA in the outer layer of both populations (Fig. 2A, left). Relative fluorescence intensity of WGA-treated cells demonstrated that external and internal cells had no significant differences in their signal levels (Fig. 2B), suggesting that enterococcal peptidoglycan and other WGA-interacting polymers (21, 30) were unaltered in both populations.

We further analyzed the E. faecalis plasma membrane using FM 5-95 and its fixable analog, FM 4-64FX, which are fluorescent lipophilic dyes that integrate into the outer leaflet of biological membranes (31–34). E. faecalis from the colony-print exhibited greater FM fluorescence intensity than their nonpenetrating counterparts 72 h postinoculation (Fig. 2A, center). These signals were localized in domains or distributed along the cell periphery, whereas the nonpenetrating cells showed a diffuse and faint fluorescence (Fig. 2A, center). Flow cytometric analyses using FM 4-64FX confirmed these observations (Fig. 2C and D). The population inside the agar showed an ~2-fold increase in mean fluorescence intensity compared to external bacteria, both 48 h (Fig. S2A and B) and 72 h postinoculation (Fig. 2C and D). Conversely, agar-penetrating cells collected at earlier time points (24 h) exhibited lower values than those of the nonpenetrating population (Fig. S2A and B). The FM dyes used are cationic styryl compounds with higher affinity for membranes enriched in negatively charged phospholipids (31). To evaluate whether other enterococcal phospholipids were altered during penetration, we used boron-dipyrromethene 493/503 (BODIPY), which preferentially binds to neutral lipids in the cell (35–37). Consistent with our results using FM dyes, CLSM experiments determined that cells penetrating the agar surface for 72 h had increased BODIPY staining compared to the external population (Fig. 2A, right). Kinetic analyses of BODIPY staining using flow cytometry confirmed that E. faecalis organisms from the colony-print exhibited significantly more fluorescence than their nonpenetrating counterparts at all time points (Fig. 2E and F; Fig. S2C and D). Hence, surface penetration elicits alterations in neutral and anionic lipids that might support enterococcal CE modifications.

10.1128/mbio.02294-22.2 FIG S2 Surface penetration elicits lipid accumulation in E. faecalis cells. (A to D) Flow cytometry-based analyses of nonpenetrating (outside) and agar-penetrating (inside) enterococci stained with lipid-specific dyes FM 4-64FX (515/640; A and B) or BODIPY (493/503; C and D). Data are presented as mean fluorescence intensity (MFI; A and C), and corresponding histograms of one representative replicate are shown (B and D). Unstained cells were used as the intrinsic background control (Ct). Two-way ANOVA (Tukey’s test) was used for panels A and C; *n* = 3. ns, not significant. Each data point represents an independent biological replicate. Download FIG S2, TIF file, 0.6 MB. Copyright © 2022 Ramos et al. 2022 Ramos et al. https://creativecommons.org/licenses/by/4.0/ This content is distributed under the terms of the Creative Commons Attribution 4.0 International license.

### Increased membrane glycolipid content in surface-penetrating enterococci.

**FIG 3:**
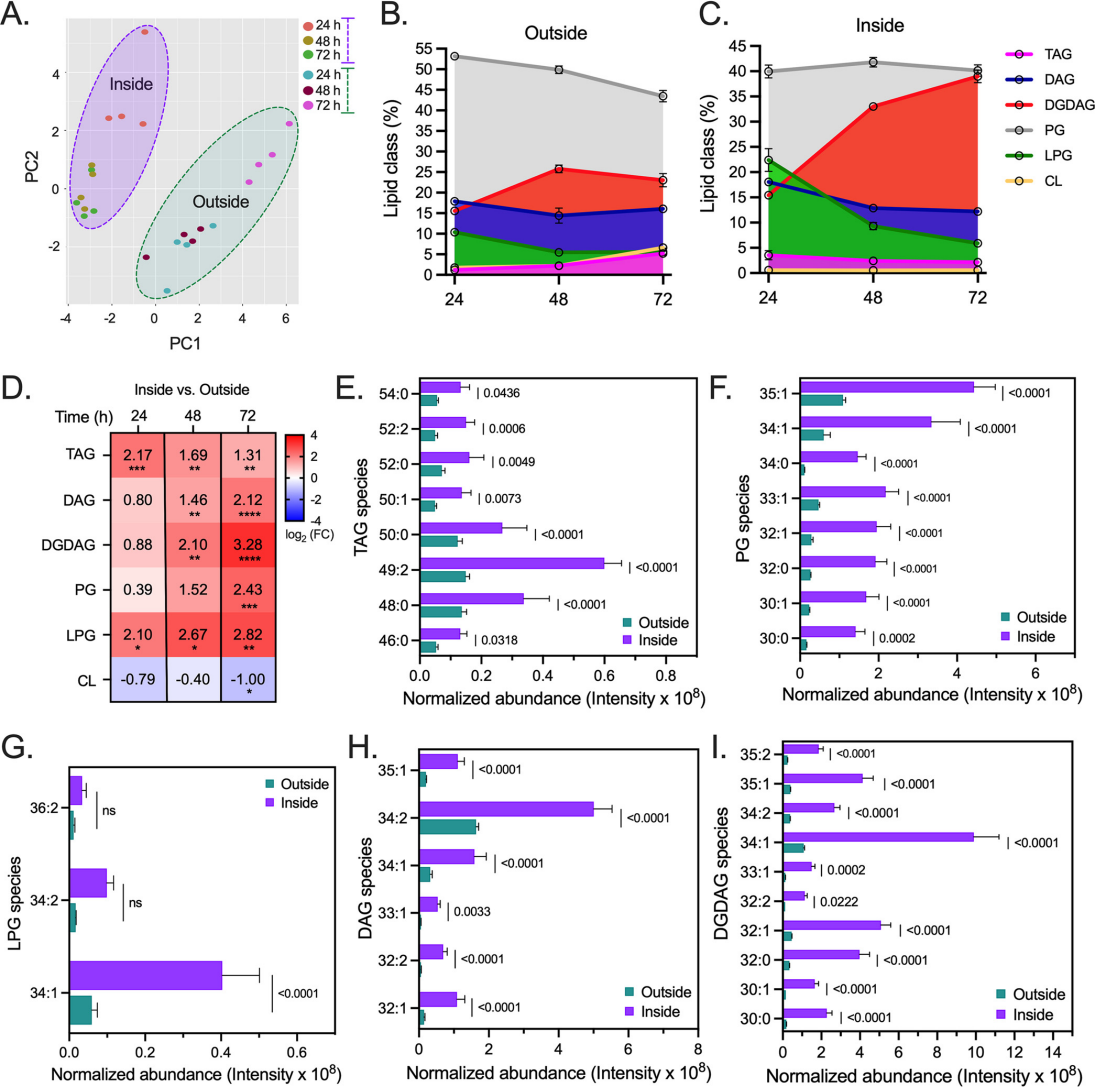
Surface penetration by E. faecalis is accompanied by marked variations in its phospholipids and phosphorus-free lipid composition. (A to I) Lipidomic analyses of external (outside) and agar-penetrating (inside) enterococcal cells grown on MOLP over time. (A) Principal-component analysis (PCA) of the total lipid classes obtained for both populations. (B and C) Percentage of the relative abundance of triacylglycerol (TAG), diacylglycerol (DAG), diglucosyl-diacylglycerol (DGDAG), phosphatidylglycerol (PG) lysyl-phosphatidylglycerol (LPG), and cardiolipin (CL) detected in nonpenetrating (B) and surface-penetrating (C) enterococci. All classes were normalized to total protein content. (D) Heatmaps depicting the log_2_ fold change (FC) of each lipid class in internal versus external populations. Two-way ANOVA (Tukey’s test); (*, *P* < 0.05; **, *P* < 0.01; ***, *P* < 0.001; ****, *P* < 0.0001). (E to I) The levels of lipid species differentially produced by both populations 72 h postinoculation are shown as normalized abundance ([intensity/total protein]/10^8^) per fatty acyl composition (number of carbon atoms and saturation). All data are presented as the mean ± standard error (SE); *n* = 4; two-way ANOVA (Šídák’s test) was used for panels (E to I); ns, not significant.

The membrane lipid bilayer is formed by phospholipids formed by a head group, two fatty acids, a glycerol moiety, and a phosphate group (38, 39). E. faecalis produces copious amounts of membrane phospholipids and phosphorus-free lipids, such as diacylglycerol (DAG) and glycolipids (40–45). We profiled the membrane lipid composition of external versus agar-penetrating bacteria using lipidomic analysis of lipid classes commonly detected in E. faecalis (40–45). Principal-component analysis (PCA) of the lipids detected clearly distinguished each population (Fig. 3A), suggesting that their lipid profiles differed drastically during surface penetration. Both surface-penetrating and external cells produced anionic phosphatidylglycerol (PG), cardiolipin (CL), cationic lysyl-phosphatidylglycerol (LPG), neutral DAG, triacylglycerol (TAG), and diglucosyl-diacylglycerol (DGDAG) lipid classes (Fig. 3B and C). As previously reported (40, 41), the lipid profile of strain OG1RF was dominated by PG (39 to 53% of all lipid classes), regardless of surface localization or time point of analysis (Fig. 3B and C). However, 72 h after surface inoculation, the levels of PG significantly decreased in external cells (Fig. 3B and Table S1) while remaining steady in the surface-penetrating population (Fig. 3C). Other predominant lipid classes detected were DAG and the glycolipid DGDAG. In E. faecalis cells on the agar (outside), DAG remained constant (14 to 18% of total lipid classes) and DGDAG reached ~23% at 72 h (Fig. 3B). Surprisingly, these lipids exhibited contrasting profiles in surface-penetrating enterococci: while DAG abundance decreased from 18 to ~12%, DGDAG increased progressively to reach ~39% of the total lipid classes at 72 h, becoming as abundant as PG in this population over time (Fig. 3C and Table S1). CL, LPG, and TAG were also detected, albeit at lower levels, in both populations throughout the experiment (Fig. 3B and C). External cells slightly increased TAG and CL while reducing their LPG content at later time points (Fig. 3B and Table S1). Agar-penetrating enterococci also showed a significant decrease in LPG abundance, but their TAG and CL levels remained unchanged (Fig. 3C and Table S1).

10.1128/mbio.02294-22.8 TABLE S1 Statistical analysis of membrane lipid classes differentially produced by Enterococcus faecalis during surface penetration. Download Table S1, PDF file, 0.02 MB. Copyright © 2022 Ramos et al. 2022 Ramos et al. https://creativecommons.org/licenses/by/4.0/ This content is distributed under the terms of the Creative Commons Attribution 4.0 International license.

Regarding relative abundance compared with their external counterparts, agar-penetrating bacteria demonstrated increased TAG, DAG, DGDAG, PG, and LPG content, especially at later time points (Fig. 3D). These results were consistent with our initial observations using membrane fluorescent dyes, denoting augmented anionic (PG) and neutral (DAG, TAG, and DGDAG) lipids in agar-penetrating enterococci (Fig. 2). Next, we assessed changes in lipid species within the classes identified in E. faecalis growing inside or outside the semisolid surface for 72 h, as we observed a significant difference between the two populations at this time point. Out of 60 TAGs identified, 8 species (5 saturated and 3 unsaturated) were significantly increased in the internal population (Fig. 3E). Of the 26 PGs detected, 3 saturated and 5 monounsaturated species were enriched in the internal population (Fig. 3F). PG can be modified with positively charged amino acids, such as lysine, to produce LPG (46). Of the three LPG species identified, only LPG 34:1 was significantly increased in the surface-penetrating population (Fig. 3G). Our assays further identified 35 DAG and 32 DGDAG species in both E. faecalis populations. Of these, 8 DAGs (Fig. 3H) and 10 DGDAGs (Fig. 3I), mostly unsaturated species, were significantly elevated in agar-penetrating cells. Enterococci synthesize DGDAG by enzymatically transferring two UDP-glucose onto DAG (47, 48). Notably, 8 DGDAGs predominant in internal cells had the same number of fatty acyl carbon atoms and bonds as the DAGs that were elevated in the same population (Fig. 3H and I), suggesting that E. faecalis inside the agar might use these unsaturated DAGs as substrates to produce and accumulate higher levels of DGDAGs.

Taken together, these findings demonstrate that surface penetration by E. faecalis is accompanied by marked alterations in its glycolipid composition, and these changes may help penetrating cells to strengthen their membranes as an adaptive process.

### Surface penetration induces gene programs implicated in CE synthesis and metabolism.

**FIG 4:**
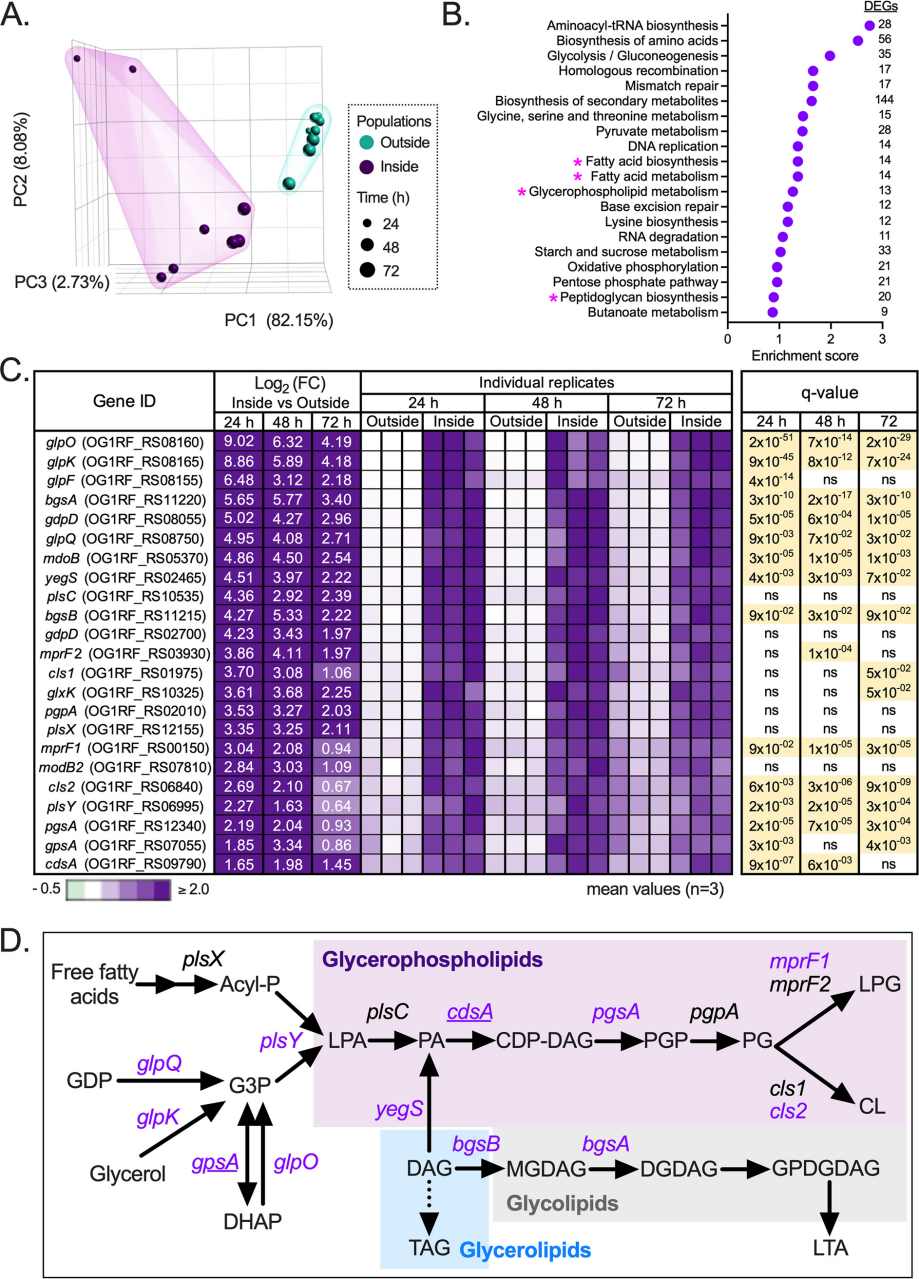
Surface penetration induces gene programs involved in CE synthesis and lipid metabolism. (A to C) RNA sequencing analysis of E. faecalis grown on MOLP shows marked differences in global gene expression profiles according to their surface localization over time. (A) PCA denoting segregation of nonpenetrating (outside) and agar-penetrating (inside) enterococcal populations. (B) Top 20 enriched pathways obtained from differentially expressed genes (DEGs) in E. faecalis penetrating the semisolid surface for 24 h, based on the KEGG database. CE biogenesis-related metabolic pathways are highlighted (magenta asterisks). (C) Top 23 differentially expressed transcripts in internal (inside) versus external (outside) enterococci involved in glycerophospholipid and glycolipid biosynthesis. Individual replicate heatmaps show the log_2_ fold change (FC) of normalized transcripts from inside versus outside samples. Gene expression values were normalized to the reference, which is the mean across inside or outside samples for each time point. Significant transcripts according to *P* values adjusted to the false-discovery rate (*q* value, <0.05) are highlighted in yellow. (D) Bacterial metabolic pathways and corresponding gene homologs significantly overexpressed (purple) in the penetrating population compared with cells on the surface. Biosynthesis of glycerophospholipids (purple shading) requires glycerol-3-phospate (G3P), which could be obtained from either glycerone-phosphate (GDP), dihydroxyacetone phosphate (DHAP), or glycerol. Acyl-phosphate (Acyl-P), an end product from fatty acid synthesis, can be used as an acyl donor to produce lysophosphatidic acid (LPA) followed by phosphatidic acid (PA) from G3P. PA is a substrate for CDP-diacylglycerol (CDP-DAG), which is used for synthesis of phosphatidylglycerol phosphate (PGP), and this, in turn, is transformed into the anionic lipid PG. Then, PG can be converted to cationic LPG in a one-step reaction, or two molecules of PG can react, forming CL. The biogenesis of glycolipids (gray shading) starts with the transfer of a sugar monomer from activated sugars such as UDP-glucose to DAG, leading to the generation of monoglucosyl-diacylglycerol (MGDAG). Subsequently, additional sugars can be transferred to the MGDAG, leading to more complex glycolipids such us DGDAG or glycerophospho-diglucosyl-diacylglycerol (GPDGDAG). The latter is a phosphoglycolipid that works as an intermediate in the synthesis of lipoteichoic acid (LTA), which consists of polyglycerophosphate polymers anchored to the membrane by glycolipids. The transfer of glycerophosphate units from PG during LTA biosynthesis releases the neutral glycerolipid, DAG (blue shading), which could be recycled into glycolipid and phospholipid metabolism or converted to triacylglycerol (TGA). Genes significantly upregulated at only one time point during the experiment are underlined. The dotted line denotes a putative reaction in E. faecalis. For a more detailed description of these pathways see references 39, 47–49, 69, 80, and 123–126.

We examined whether the CE lipid alterations identified were associated with transcriptional reprogramming during the penetration process. Using RNA sequencing (RNA-seq), we compared the global transcriptomic profile of E. faecalis cells growing outside or inside the agar 24, 48, and 72 h after surface inoculation. Of note, PCA denoted again a clear segregation of E. faecalis gene expression profiles, depending on the bacterial localization in the medium (Fig. 4A). We identified 1,429 genes whose expression was significantly altered between surface-penetrating and nonpenetrating cells throughout the experiment. KEGG-based enrichment pathway analysis of differentially expressed transcripts between both populations revealed enrichment in pathways involved in amino acid, nitrogen, nucleotide, and carbon metabolism, CE biogenesis (lipid and glycan metabolism), signal transduction, and membrane transport, among others (Fig. 4B and Fig. S3). Within CE biosynthesis, 128 transcripts were differentially expressed in internal versus external enterococci (Table S2). From those, 23 transcripts were associated with glycerophospholipid and glycolipid biosynthetic pathways (Fig. 4C and D). The *glpKOF* regulon was selectively upregulated (~2- to 9-fold) in agar-penetrating enterococci throughout the course of the experiment (Fig. 4C and D). This regulon comprises an operon encoding three proteins (GlpK, GlpO, and GlpF) that are implicated in the synthesis of glycerol-3-phosphate (G3P), a central precursor of phospholipids (Fig. 4D), and a source to generate ATP in several bacteria (49). Moreover, our RNA-seq analysis revealed that surface penetration increased the expression of multiple genes coding enzymes involved in bacterial glycerophospholipid synthesis (Fig. 4C and D), including homologs for *plsY* (coding a putative enzyme part of the glycerol-phosphate acyltransferase system), *cdsA* (cytidyltransferase), *pgsA* (phosphatidyltransferase), *yegS* (encoding a putative DAG kinase), *cls2* (cardiolipin synthase 2), and *mprF1* (multiple resistance factor 1) loci (39, 40, 50–53).

10.1128/mbio.02294-22.3 FIG S3 Biological pathways enriched in E. faecalis during agar penetration. (A) Metabolic categories for differentially expressed genes (DEGs) enriched between agar-penetrating (inside) and nonpenetrating (outside) enterococci 24 h postinoculation. (B and C) Top 20 enriched pathways from the categories shown in panel A of DEGs during E. faecalis surface penetration at 48 h (B) and 72 h (C) based on the Kyoto Encyclopedia of Genes and Genomes (KEGG) database. CE biogenesis-related metabolic categories/pathways are highlighted (magenta asterisks). Download FIG S3, TIF file, 0.5 MB. Copyright © 2022 Ramos et al. 2022 Ramos et al. https://creativecommons.org/licenses/by/4.0/ This content is distributed under the terms of the Creative Commons Attribution 4.0 International license.

10.1128/mbio.02294-22.9 TABLE S2 Cell envelope biogenesis-related transcripts differentially expressed in internal versus external enterococci. Download Table S2, PDF file, 0.08 MB. Copyright © 2022 Ramos et al. 2022 Ramos et al. https://creativecommons.org/licenses/by/4.0/ This content is distributed under the terms of the Creative Commons Attribution 4.0 International license.

Corresponding with our initial lipidomic results (Fig. 3D to I), transcripts involved in the synthesis of DGDAG (54, 55), were also upregulated in the surface-penetrating population. Specifically, expression of *bgsA* (biofilm-associated glycolipid synthesis) encoding a 1,2-glucosyl transferase (54) was significantly elevated (~5.6-, ~5.7-, and ~3.4-fold at 24, 48, and 72 h, respectively) in the surface-penetrating population compared with enterococci remaining outside MOLP (Fig. 4C and D). This enzyme catalyzes the glycosylation of monoglucosyl-diacylglycerol (MGDAG) to yield DGDAG in enterococci and other Gram-positive bacteria (54, 55). *bgsB*, which is located immediately downstream of *bgsA*, was also upregulated (~4.3-, ~5.3-, and ~2.2-fold at 24, 48, and 72 h, respectively) during enterococcal agar penetration (Fig. 4C to D, inside). BgsB is a 1,2-diacylglycerol 3-glucosyltransferase responsible for the glycosylation of DAG to form MGDAG (Fig. 4D), the first step in glycolipid synthesis in E. faecalis (48). Real-time quantitative PCR (RT-qPCR) analyses confirmed higher expression of *bgsA*, *bgsB*, *glpK*, and *mprF1* by agar-penetrating E. faecalis throughout the experiment (Fig. S4).

10.1128/mbio.02294-22.4 FIG S4 Expression of genes encoding proteins involved in CE-related metabolic pathways. (A to D) RT-qPCR analysis for the expression of biofilm-associated glycolipid synthesis *bgsB* (A), *bgsA* (B), glycerol kinase (*glpK*; C), and multiple resistance factor 1 (*mprf1*; D) in nonpenetrating (outside) and agar-penetrating enterococcal cells (inside) at 24, 48, and 72 h postinoculation on MOLP. Transcripts were normalized to endogenous *recA* expression. All data are shown as the mean ± SE; *n* = 3; unpaired *t* test. Download FIG S4, TIF file, 0.4 MB. Copyright © 2022 Ramos et al. 2022 Ramos et al. https://creativecommons.org/licenses/by/4.0/ This content is distributed under the terms of the Creative Commons Attribution 4.0 International license.

These results reveal that surface penetration induces marked transcriptional reprogramming in E. faecalis, which favors the upregulation of genes involved in CE biogenesis and glycolipid metabolism.

### Surface penetration promotes E. faecalis tolerance to CE-targeting antimicrobials.

**FIG 5:**
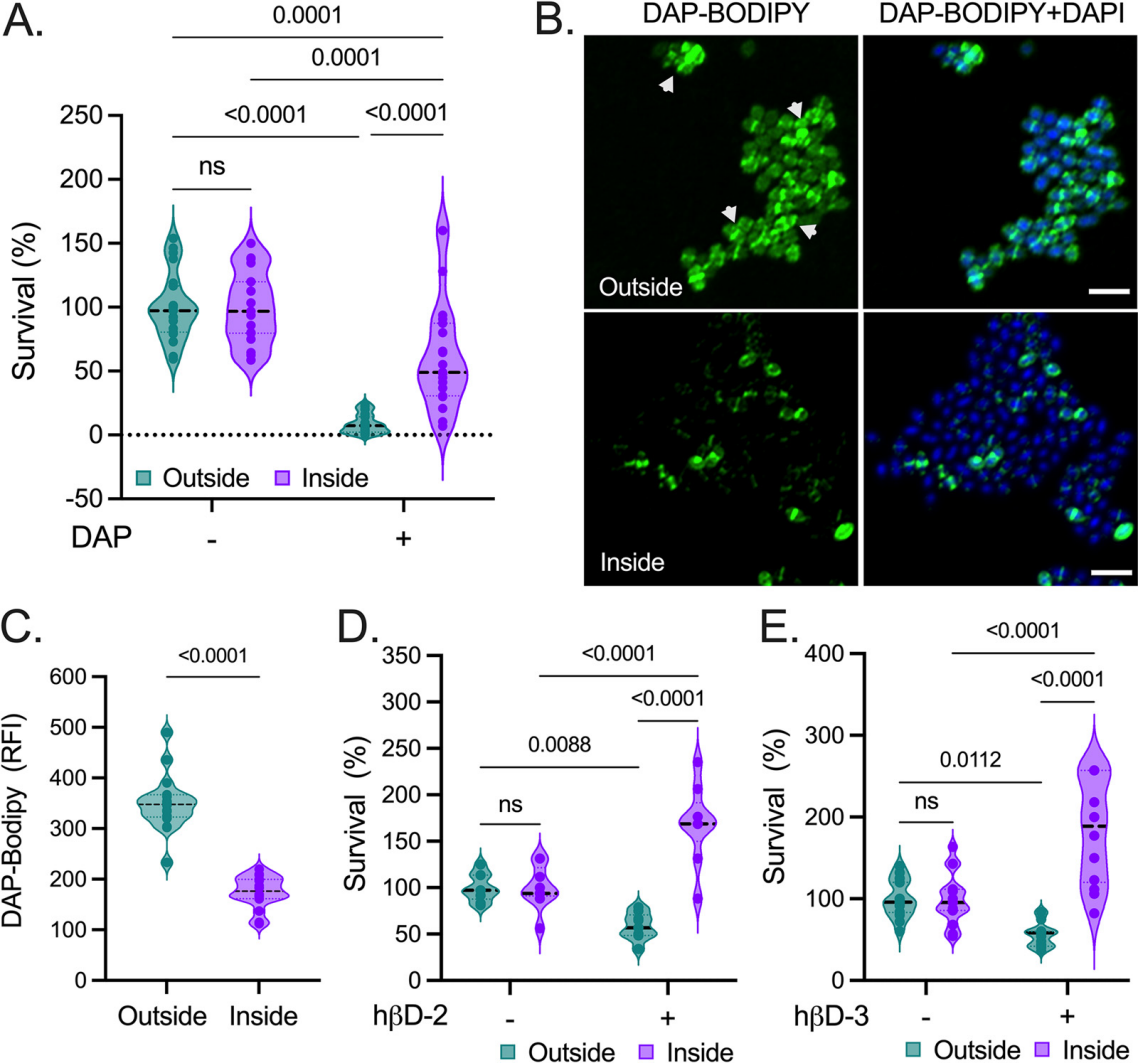
Surface penetration enhances enterococcal tolerance to CE-targeting antimicrobials. (A, D, and E) Bacteria were recovered from inside the agar or the surface (outside) 72 h after inoculation on MOLP, and cells were then exposed to the indicated envelope stressors for 2 h. (A) Percent survival (%) calculated after treatment with 4 mg/mL of daptomycin (DAP). Data are presented as the mean ± SE of 7 independent assays with 3 to 4 biological replicates; *n* = 23. (B) CLSM images of external (top) and internal (bottom) E. faecalis cells grown on MOLP for 72 h and stained with DAP-conjugated BODIPY-FL (DAP-BODIPY; green). Scale bar = 2 μm. (C) Relative fluorescence intensity (RFI) of DAP-BODIPY-stained enterococci quantified using Fiji (*n* = 18). Percent survival (%) of enterococcal cells exposed to 0.6 μg/mL of human β-defensins (hβDs), hβD-2 (D), or hβD-3 (E). Data represent either two (D) or three (E) independent assays; *n* = 18. Each point denotes an independent biological replicate. Two-way ANOVA (Šídák’s test) was used for panels (A, D, and E). Two-tailed unpaired Wilcoxon-Mann-Whitney test was used for panel C. ns, not significant.

Alterations in membrane lipid levels have been implicated in sensitivity to the antibiotic daptomycin (DAP) in multiple bacteria (43, 56–58). Since we found upregulation of genes coding for enzymes involved in glycolipid synthesis (Fig. 4C and D) and increased DGDAG levels (Fig. 3D and I) in agar-penetrating E. faecalis, we evaluated the sensitivity of this population to DAP exposure. The growth of external and agar-penetrating cells was comparable in the absence of DAP. However, bacteria recovered from the internal colony-print demonstrated an ~6.9-fold increase in their basal tolerance to this antibiotic compared with external cells (Fig. 5A). Since surface penetration promotes the formation of multicellular aggregates (21, 59), this trait might contribute to the antimicrobial tolerance observed in penetrating cells. However, disaggregation by mild sonication did not compromise the enhanced DAP tolerance observed in the surface-penetrating population (Fig. S5 and Fig. 5A). Next, using DAP conjugated to BODIPY-FL (DAP-BODIPY) (60, 61), we observed that nonpenetrating bacteria showed strong fluorescence in the division septum as well as peppering patterns at the cell periphery (Fig. 5B, top). In contrast, E. faecalis cells inside the agar showed a significant reduction in DAP-BODIPY signal compared with their external counterparts (Fig. 5B, bottom, and Fig. 5C).

10.1128/mbio.02294-22.5 FIG S5 Role of cellular aggregates in the tolerance to daptomycin. 72-h-MOLP-grown E. faecalis cells from inside the agar or the colony on the surface (outside) were sonicated to disaggregate cells. The percent (%) survival relative to unexposed (without daptomycin, DAP) enterococci was calculated after 2 h of treating both populations with and without 4 mg/mL of DAP. Data represent three independent assays; *n* = 11. Two-way ANOVA (Tukey’s test); ns, not significant. Download FIG S5, TIF file, 0.1 MB. Copyright © 2022 Ramos et al. 2022 Ramos et al. https://creativecommons.org/licenses/by/4.0/ This content is distributed under the terms of the Creative Commons Attribution 4.0 International license.

DAP is a cyclic lipopeptide that, once complexed with calcium, shares several properties with cationic antimicrobial peptides (CAMPs) (62). Therefore, we tested whether E. faecalis penetration could promote tolerance to CAMPs normally synthesized in the GIT, such as human β-defensins (hβDs) that are expressed by various epithelial cells (63, 64). Exposure to either hβD-2 or hβD-3 reduced the viability of nonpenetrating E. faecalis cells by ~50% (Fig. 5D and E), whereas cells recovered from the colony-print exhibited an ~3-fold increase in their tolerance to both CAMPs compared with their external counterparts (Fig. 5D and E). Surprisingly, surface-penetrating cells exposed to hβDs nearly doubled their density in comparison with their untreated counterparts (Fig. 5D and E). Hence, CE alterations evoked during surface penetration, such as increased glycolipid production, might endow E. faecalis cells with increased tolerance to DAP and other CAMPs.

### BgsB is necessary for CE remodeling during surface penetration.

**FIG 6:**
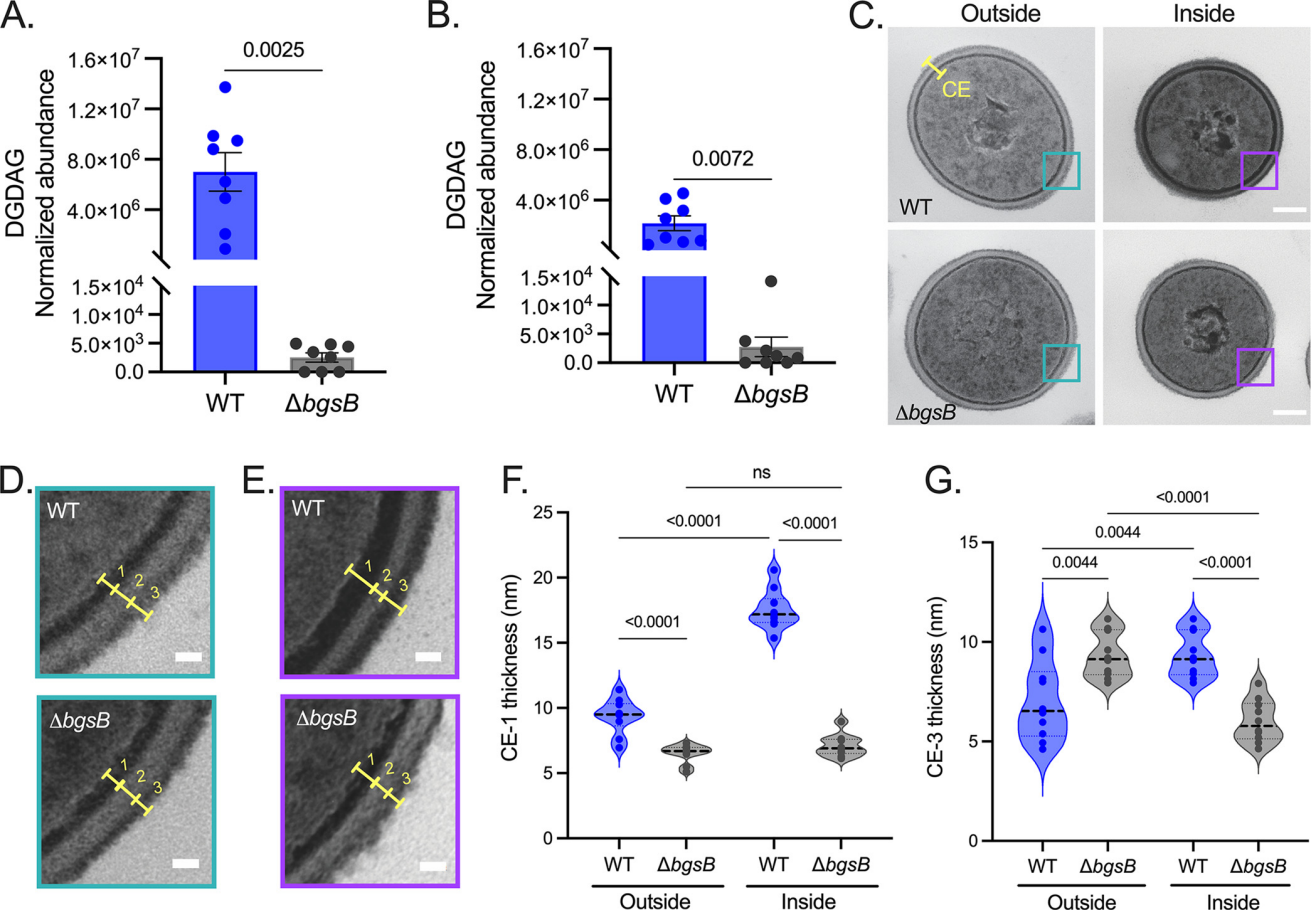
Remodeling of the enterococcal CE during surface penetration requires the glucosyltransferase BgsB. (A and B) The abundance of DGDAG (intensity/total protein) was determined by LC-MS analyses in both E. faecalis WT and Δ*bgsB* agar-penetrating (A) and nonpenetrating (B) cells 72 h postinoculation. Data are shown as the mean ± SE from two independent assays; *n* = 8. (C) Electron micrographs of external (outside) and agar-penetrating (inside) enterococcal strains 72 h postinoculation on MOLP. The yellow bracket denotes the bacterial CE. Scale bar = 100 nm. (D and E) Cyan and purple squares indicate the magnified areas of TEM images in panel C of E. faecalis WT and Δ*bgsB* cells obtained from the surface (D) or inside the agar (E). A three-zone CE (yellow brackets) characterized by an inner electron-dark zone (1), followed by an intermediate electron-translucent area (2) and an outermost fibrous heavily stained layer (3) is highlighted (D and E). (F and G) Quantification of thickness of CE electron-dark zones 1 (F) and 3 (G) reveals significant differences in these structures between mutant and parent strains (*n* = 10). Each point denotes an independent biological replicate. Unpaired *t* test (Welch’s test) and two-way ANOVA (Šídák’s test) were used for panels A and B and D and E, respectively. ns, not significant. Scale bars = (D) 100 nm and (E and F) 15 nm.

Diacylglycerol glucosyltransferases, such as BgsB, are required for the first reaction of glycolipid biogenesis in E. faecalis 12030 (48) and other Gram-positive bacteria (55). Hence, to functionally define the role of enhanced glycolipid synthesis in E. faecalis CE remodeling during surface penetration, we tested a mutant strain lacking *bgsB* (65). Liquid chromatography-mass spectrometry (LC-MS) analyses confirmed that agar-penetrating enterococci had higher levels of DGDAG (~3.3-fold increase) than external cells and that loss of *bgsB* ablated this lipid class in both enterococcal populations (Fig. 6A and B). Consistent with our initial observations, TEM analyses showed that wild-type (WT) cells inside the agar 72 h postinoculation demonstrated increased CE thickness (Fig. 6C to E; Fig. S6A), where zones CE-1 and CE-3 were thicker than in their external counterparts (Fig. 1D to G; Fig. 6D to G). Loss of BsgB markedly affected the CE remodeling observed in agar-penetrating cells (Fig. 1B to G and Fig. 6D to G; Fig. S6A), as evidenced by a significant reduction in both CE-1 and CE-3 compared with their WT counterparts (Fig. 6D to G). No significant differences in the total area of the CE-2 region were observed in agar-penetrating or external cells of either genotype (Fig. S6B and C). Therefore, BgsB is necessary for the increase in glycolipid levels and CE thickening elicited during surface penetration.

10.1128/mbio.02294-22.6 FIG S6 Ultrastructural features and sensitivity to envelope stressors in WT and Δ*bgsB* E. faecalis during surface penetration. (A to C) TEM images for agar-penetrating (inside) and nonpenetrating (outside) cells of both enterococcal strains were analyzed using Fiji to determine (A) CE thickness, (B) cell area, and (C) width of CE electron-translucent zone 2 (CE-2, depicted in Fig. 6). Data points represent independent biological replicates; *n* = 20 for panels A and B and *n* = 10 for panel C; two-way ANOVA (Šídák’s test). (D to F) Bacteria were recovered from the agar surface (nonpenetrating) 72 h postinoculation on MOLP, and the percent (%) survival was calculated after 2 h of treatment with 4 mg/mL DAP (D), 0.6 μg/mL hβD-2 (E), or 0.6 μg/mL hβD-3 (F). Data represent three independent assays (*n* = 9) for panel D and two independent experiments (*n* = 12) for (panels E and F). Two-way ANOVA (Tukey’s test) was used for all data; ns, not significant. Download FIG S6, TIF file, 0.3 MB. Copyright © 2022 Ramos et al. 2022 Ramos et al. https://creativecommons.org/licenses/by/4.0/ This content is distributed under the terms of the Creative Commons Attribution 4.0 International license.

### Loss of BgsB sensitizes surface-penetrating enterococci to membrane-damaging agents.

**FIG 7:**
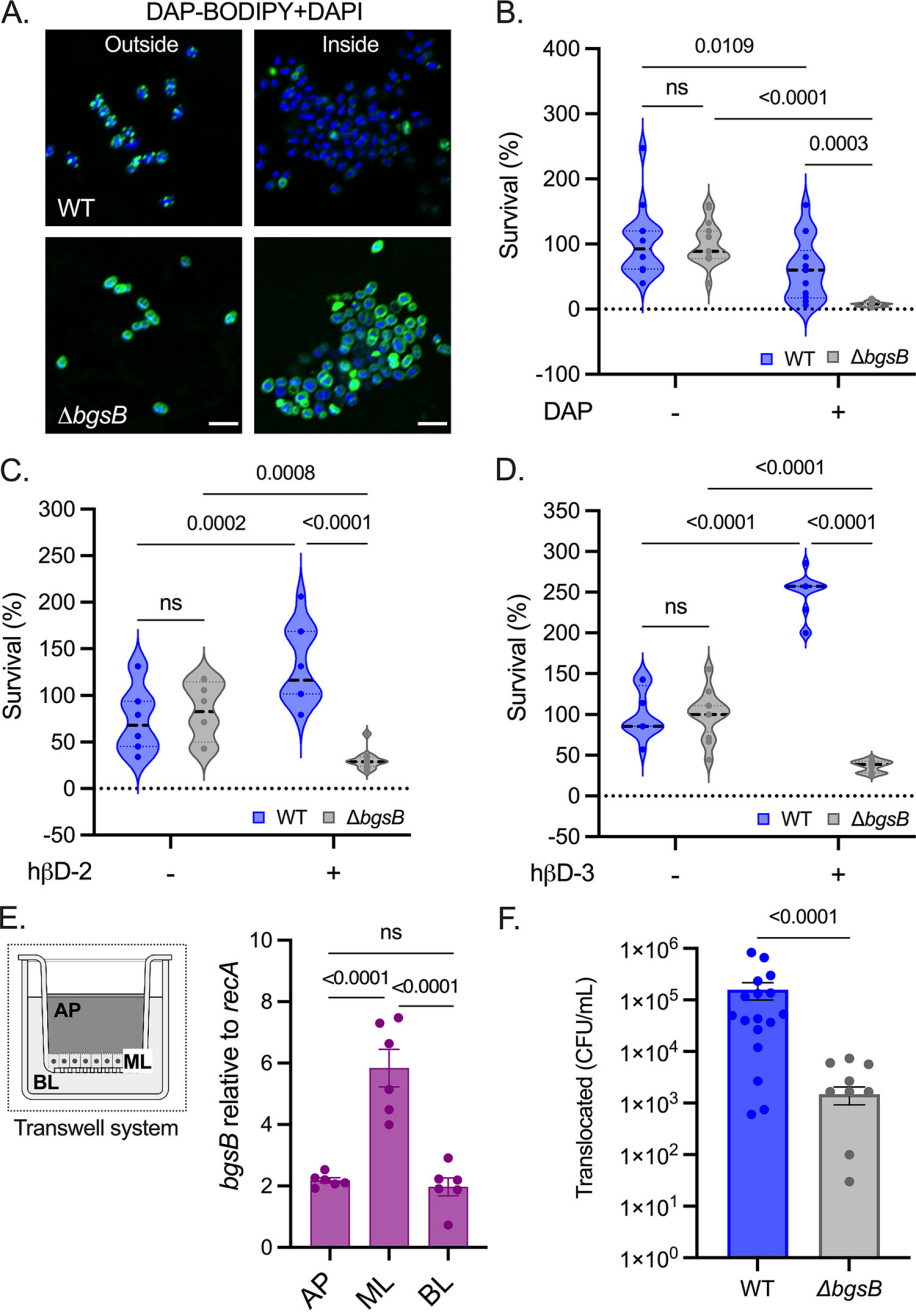
Loss of BgsB impairs E. faecalis tolerance to membrane-damaging agents and reduces its translocation capacity. (A) Overlayed CLSM-images of E. faecalis WT (top) and Δ*bgsB* (bottom) inside (left) the agar or on the surface (outside; right) stained with DAP-BODIPY to identify sites of DAP interaction with the plasma membrane (green) and DAPI for visualization of DNA (blue). Scale bar = 2 μm. (B to D) Percent survival (%) of agar-penetrating populations treated for 2 h with 4 mg/mL DAP (B), 0.6 μg/mL hβD-2 (C), or 0.6 μg/mL hβD-3 (D). Data represent three independent assays (*n* = 9) for panel B and two independent experiments (*n* = 12) for (panels C and D). (E) Scheme of the two-chamber transcytosis system is shown (left), wherein the apical side (AP), the epithelial cell monolayer (ML), and the basolateral side (BL) are highlighted; (right) RT-qPCR analysis of E. faecalis *bgsB* expression 4 h postcoculture with intestinal epithelial cells. Data are normalized to endogenous *recA* expression (mean ± SE; *n* = 6). (F) E. faecalis WT and Δ*bgsB* translocation through T84 human intestinal epithelial cell monolayers. CFU/mL of viable bacterial cells that pass through the ML to the BL after 6 h of incubation (translocated). Data are presented as the mean ± SE of three independent experiments; *n* = 18. Two-way ANOVA (Tukey’s test) was used for panels B to E. Two-tailed unpaired Wilcoxon-Mann-Whitney test was used for panel F. ns, not significant.

Enterococci that successfully penetrated the semisolid agar surface showed superior tolerance to DAP and CAMPs compared to external cells (Fig. 5). We assessed whether BgsB mediated this major adaptive process. CLSM analyses showed that agar-penetrating cells devoid of BgsB had increased DAP-BODIPY binding compared with their WT counterparts (Fig. 7A). While WT and Δ*bgsB* cells remaining on the agar surface exhibited comparable sensitivity to DAP (Fig. S6D), Δ*bgsB* cells inside the agar demonstrated a 7.6-fold reduction in DAP tolerance compared with their WT counterparts (Fig. 7B). Furthermore, Δ*bgsB* cells recovered from inside the agar showed augmented sensitivity to both hβD-2 and hβD-3 compared to the parent strain in the same location (Fig. 7C and D), whereas external cells from both genotypes exhibited similar sensitivity to these CAMPs (Fig. S6E and F). Taken together, these data indicate that BgsB-driven CE remodeling is necessary for the increased tolerance to membrane-damaging agents observed in the agar-penetrating community.

### BgsB deficiency compromises E. faecalis migration through surfaces.

We reported that E. faecalis translocates through human intestinal cell monolayers using mechanisms similar to those mediating semisolid surface penetration (21). Hence, we hypothesized that BgsB could be required for E. faecalis translocation across host intestinal epithelial barriers that normally produce CAMPs. To test this, we used a two-chamber transcytosis system (21, 22) in which enterococci inoculated on the apical side translocate across T84 human intestinal cell monolayers to reach the basolateral side of the chamber (Fig. 7E, left). RT-qPCR analyses of cells recovered 4 h postinoculation revealed marked *bgsB* (Fig. 7E, right) and *bgsA* (Fig. S7A) overexpression by enterococci attached to and passing through the epithelial monolayers. We found that 6 h postinfection, the monolayers exhibited transepithelial resistance values similar (~1,478 Ω/cm2) to those obtained prior to bacterial inoculation (~1,319 Ω/cm2), indicating that the T84 cell monolayers remained mostly intact throughout the experiment. All strains evaluated reached approximately 10^8^ to 10^9^ CFU/mL on the apical side of all wells tested (Fig. S7B). In sharp contrast to WT OG1RF, which demonstrated a remarkable capacity to translocate in this assay, Δ*bgsB* showed a significant decrease of 2 orders of magnitude in the number of CFU detected on the basolateral side (~1.6 × 10^5^ and ~1.5 × 10^3^ CFU/mL for WT and mutant, respectively; Fig. 7F). Of note, the absence of BgsB also impaired the ability of E. faecalis to penetrate agar surfaces, consistent with the visible decrease in the colony-print generated by Δ*bgsB* (Fig. S7C, bottom) and the one-log reduction in CFU obtained for this mutant’s agar-penetrating population, compared with the parent strain (Fig. S7C, top). The development of external colonies by Δ*bgsB* was not different from that of WT, as evidenced by the similar CFU recovered for both strains 72 h postinoculation on the surface of MOLP (Fig. S7D). However, Δ*bgsB* had a slight growth difference compared to WT when grown on liquid MOLP for 24 h (Fig. S7E). Thus, the enhanced synthesis of glycolipids driven by BgsB is required for efficient E. faecalis semisolid surface penetration and translocation through host intestinal epithelial barriers.

10.1128/mbio.02294-22.7 FIG S7 Analysis of E. faecalis WT and Δ*bgsB* during penetration/translocation. (A) RT-qPCR analysis of *bgsA* expression in enterococcal cells after a 4-h coculture with T84 human intestinal epithelial cells. Bacteria were collected from the medium at the apical side (AP), the epithelial cell monolayer (ML), and the basolateral side (BL) of a two-chamber transcytosis system (scheme shown in Fig. 7). Data are normalized to *recA* expression (mean ± SE; *n* = 6). (B) CFU/mL of viable bacterial cells recovered from the medium at the AP after 6 h of translocation through intestinal epithelial monolayers (nontranslocated). Data are presented as the mean ± SE of three independent experiments; *n* = 18. (C and D) Capacity of E. faecalis of the indicated genotypes to penetrate MOLP, determined by quantifying CFU/mL inside (C) or outside (D) the agar 72 h postinoculation. When strains exhibited growth differences, the final CFU/mL were normalized to the initial absorbance (OD_600_) of each saline suspension from which serial dilutions were performed (normalized CFU/mL). Data are presented as the mean ± SE of two independent assays; *n* = 6. Scale bar = 1500 μm. (E) E. faecalis WT and Δ*bgsB* were grown in MOLP broth for 24 h at 37°C. Bacterial growth was determined by measuring the absorbance at 600 nm at different time points (mean ± SE; *n* = 6). Two-way ANOVA (Tukey’s test) was used for panel A. The two-tailed unpaired Wilcoxon-Mann-Whitney test was used for panel B. The unpaired *t* test (Welch’s test) was used for (panels C and D). ns, not significant. Download FIG S7, TIF file, 0.5 MB. Copyright © 2022 Ramos et al. 2022 Ramos et al. https://creativecommons.org/licenses/by/4.0/ This content is distributed under the terms of the Creative Commons Attribution 4.0 International license.

## DISCUSSION

In this study, we present experimental evidence indicating that surface penetration promotes the generation of a distinct E. faecalis population harboring major CE lipidic and ultrastructural modifications that confer enhanced resistance to antibiotics and host-derived CAMPs. Bacteria can adapt to heterogenous microenvironments by modifying their lipid composition, allowing fine-tuning of their membrane biophysical properties (66–70). However, few studies have focused on the lipidic membrane adaptation occurring when enterococci are grown as multicellular aggregates. Our prior work determined that E. faecalis surface penetration is accompanied by the assembly of exopolysaccharide-covered aggregates (21). Here, we report that agar-penetrating enterococci exhibit marked lipid alterations with shifts in the proportions of membrane phospholipids and phosphorus-free lipids. Compared to external cells, enterococci penetrating the surface increased their anionic and neutral lipid content, with PG and the glycolipid DGDAG as the predominant classes at later time points. Notably, E. faecalis planktonic cultures also undergo lipidomic alterations in response to different growth conditions, such as exogenous supplementation of fatty acid-rich fluids, bile, or serum (41, 71–74). Consistent with our observations in surface-penetrating enterococci, these reports also found an increased glycolipid content, such as MGDAG (41), suggesting that boosting the synthesis of these lipids might be a common membrane adaptation strategy used by E. faecalis. Hence, the conditions E. faecalis endures during surface penetration foster CE remodeling, resulting in glycolipid-rich membranes with likely unique physicochemical properties.

Gram-positive bacteria possess a tripartite cell wall (24, 75, 76), in which the inner electron-dark area (such as CE-1; Fig. 1D), localized between the outer face of the membrane and the peptidoglycan layer (intermediate electron-translucent region), is considered the periplasm (24, 75–77). In Bacillus subtilis, this periplasmic space was shown to be formed by membrane-linked lipoteichoic acids, or LTAs (77). LTAs are tethered to the cell membrane by noncovalent anchoring with glycolipids (39, 55, 78). In the present study, we found that loss of *bgsB* in E. faecalis was sufficient to reduce the DGDAG content and thus ablate the CE ultrastructural differences between populations during surface penetration. Supporting our observations, E. faecalis 12030 lacking *bgsB* also completely lost its glycolipids from the membrane and generated structurally altered LTAs (48). Future experiments will be necessary to determine whether enterococci undergoing agar penetration overexpress *bgsB* to increase their glycolipids and carry out alterations on the membrane-anchored LTAs, promoting their CE thickening. Notably, our lipidomic analysis also revealed an ~9-fold increase on an ion corresponding to the glycolipid glycerophospho-diglucosyl-diacylglycerol (GPDGDAG) (40, 79), a precursor of LTA (80), during surface penetration. Determining how LTA structural changes or abundance may affect the physiology of the surface-penetrating population would therefore be of significant interest.

Membrane plasticity could help cells within aggregates respond more efficiently to external conditions by limiting metabolic exchanges, saving energy, and then promoting survival under detrimental or fluctuating conditions (69, 74, 81). We found that the ultrastructural and metabolic changes occurring in enterococci within the aggregates during surface penetration promoted their intrinsic tolerance to membrane-damaging agents. Lipidome alterations of DAP-resistant strains have been attributed to mutations in genes coding proteins involved in phospholipid biosynthesis (e.g., *pgsA* and *mprF*) and regulators of the cell envelope stress response (43, 44). Our results indicate that surface penetration induces marked transcriptional reprogramming in E. faecalis, which favors the shifting of enterococcal metabolism toward the synthesis of neutral lipids. This may represent a new protective strategy, as these lipids reduce the negative charge of the membrane required for interacting with DAP or CAMPs (82, 83). LPG content can also modulate bacterial sensitivity to certain CAMPs (84, 85). Hence, we cannot rule out the possibility that the increased abundance of LPG found in agar-penetrating enterococci (Fig. 3D) could also promote their tolerance to membrane-damaging agents. However, our RNA-seq analyses only demonstrated a significant upregulation in *mprF1*, which was previously suggested not to have a direct role in the synthesis of LPG (86). Decreased PG content has also been associated with a DAP-resistant phenotype in enterococci (43, 44). However, we found that the abundance of this phospholipid remained constant in agar-penetrating bacteria throughout the experiment. The d-alanylation of LTA also contributes to bacterial CAMP resistance by introducing positively charged groups into otherwise negatively charged teichoic acids (81, 87, 88). Our transcriptomic analysis showed that the expression of the *dlt* operon, responsible for the incorporation of d-alanine into LTA, was significantly higher in the agar-penetrating enterococci than in cells on the surface. Therefore, additional studies are needed to determine the contribution of d-alanylation in the elevated tolerance to envelope stressors induced during surface penetration.

Our study also revealed that cells attached to and undergoing translocation through intestinal epithelial cell monolayers *in vitro* exhibited marked *bgsB* and *bgsA* overexpression compared with their free-living counterparts, suggesting that enterococcal glycolipid-rich membranes are also enriched during translocation. Critically, abrogating glycolipid biogenesis, by deleting *bgsB*, reduced E. faecalis OG1RF semisolid surface penetration and translocation through epithelial cell monolayers in a two-chamber transcytosis system. Glycolipids are amphiphilic molecules, as they comprise hydrophilic glycosyl and lipophilic lipid residues (89, 90). Thus, an increase in BgsB-derived glycolipids might mediate enterococcal migration traits by acting as biosurfactants and reducing the tension between the substrate and the bacterial cell to permit spreading, as shown with B. subtilis over semisolid surfaces (91). Recently, a biosurfactant produced by an Enterococcus faecium was identified in planktonic cultures (92). Highlighting the importance of enterococcal glycolipids, previous studies using deletion mutants in E. faecalis 12030 demonstrated that BgsB and BgsA are also necessary for the development of polystyrene-attached multicellular structures (biofilms), adherence to Caco-2 cells, and prolonged bacteremia in a mouse model (48, 54). In this strain, the absence of *bgsA* impaired production of DGDAG (54) and altered its cell surface-proteome (93). Therefore, it would be important to determine whether the glycolipid shifts observed in the surface-penetrating population could modify the nature of the membrane-interacting proteins, which might be necessary for efficient E. faecalis translocation/penetration.

Whether the differences observed between agar-penetrating and external cells reflect a cause or an effect of surface penetration remains elusive. Our results suggest that BgsB is not responsible for initiating surface penetration by E. faecalis, as *bgsB* overexpression was only observed in cells that already penetrated the agar surface. However, testing whether BgsB overexpression in planktonic cells is sufficient to recapitulate the phenotype of the agar-invading enterococci will be of significant interest. Moreover, identifying the environmental cues that trigger surface penetration in a fraction of cells and understanding how these signals are sensed by enterococci deserves further investigation. In other nonmotile microbes, such as some fungi, the opening of mechanosensitive ion channels in response to surface contact elicits morphologic changes that enable fungal penetration of host tissues and agar surfaces (94). Mechanosensitive ion channels in bacteria open or close in response to environmental conditions that change their membrane tension, such as osmotic stress and the adhesion forces experienced following surface attachment (94, 95). Bacteria also possess envelope stress response systems to quickly protect cells from changing environmental conditions (96, 97). Once activated, these systems trigger signaling cascades that cause alterations in gene expression, possibly leading to bacterial envelope modifications (96, 97). Of note, our RNA-seq analyses identified overexpression of several genes coding cell envelope stress response components in agar-penetrating enterococci versus external cells, such as the LiaFSR and CroRS systems (98, 99). Both systems have been reported as drivers of antibiotic resistance in E. faecalis (44, 99–101). Understanding the role of mechanosensitive ion channels and envelope stress responses will help discern the molecular pathways triggering enterococcal penetration/translocation and the ensuing remodeling of their cell envelopes.

Collectively, our findings indicate that surface penetration favors the generation of a phenotypically distinct E. faecalis population that exhibits an adaptive advantage to resist membrane-damaging stress. Enterococci within these surface-invading communities reprogram their transcriptional profiles to remodel their CE lipid composition. Hence, the formation of invading aggregates may endow E. faecalis with improved capacity to adapt and persist in challenging environments such as the gut of susceptible hosts, favoring its translocation and success as a pathobiont.

## MATERIALS AND METHODS

### Bacterial strains, media, and culture conditions.

All strains used in this study are listed in Table S3 (65, 102). E. faecalis was cultured overnight at 37°C as previously reported (21). All chemicals were purchased from Sigma-Aldrich unless stated otherwise. The semisolid agar surface penetration assays were performed on modified medium optimal for lipopeptide production (MOLP) (21) solidified with agar (1.0%). MOLP-agar (0.8%) and MOLP-agarose (0.45%) were used for lipidomic and RNA-seq analyses, respectively. Images of external bacterial colonies grown on MOLP and the agar-penetrating colony-print were acquired using a Leica M205-FCA motorized stereo fluorescence microscope (Leica Microsystems, Inc.). CFU of both populations of E. faecalis were determined as reported (21).

10.1128/mbio.02294-22.10 TABLE S3 List of strains and primers used in this study. Download Table S3, PDF file, 0.02 MB. Copyright © 2022 Ramos et al. 2022 Ramos et al. https://creativecommons.org/licenses/by/4.0/ This content is distributed under the terms of the Creative Commons Attribution 4.0 International license.

Cocultures of T84 human intestinal epithelial monolayers and E. faecalis strains for translocation assays were performed as previously described (21) with minor modifications. Briefly, 12-h-grown bacterial cultures were diluted in Hank’s balanced salt solution (HBSS; without Ca^2+^ and Mg^2+^; Corning, Inc.) until an absorbance of 0.25 optical density at 600 nm (OD_600_; ~10^8^ CFU/mL) was reached and then were centrifuged and resuspended in translocation medium: advanced Dulbecco’s modified Eagle’s medium (DMEM/F-12) mixture supplemented with 5% fetal bovine serum (FBS), 10 mM HEPES buffer (pH 7), 0.007% β-mercaptoethanol, and 2 mM glutamine. Suspensions were inoculated in 8-day-grown intestinal epithelial monolayers. Translocation experiments were performed when cells reached confluence and a trans-epithelial electrical resistance of ~1,200 Ω/cm^2^ or higher. CFU of viable bacteria in the apical and basolateral sides were quantified at 0 and 6 h postinoculation. For each strain, 6 independent Transwells were used, and the experiments were repeated at least three independent times.

### Transmission electron microscopy (TEM).

Enterococcal cells recovered from either three external colonies on the surface or three agar-penetrating cells (colony-prints) were pooled and suspended in Dulbecco’s phosphate-buffered saline solution (DPBS; Corning-Cellgro), spun down, and then fixed in 0.1 M sodium cacodylate buffer solution containing 4% paraformaldehyde, 2.5% glutaraldehyde, and 0.02% picric acid at 4°C overnight (103). Samples were dehydrated, embedded, and sectioned for electron microscopy analysis following the standard protocol at Weill Cornell CLC Imaging Core Facility (104). Sections were viewed on a JEM-1400 transmission electron microscope (JEOL) operated at 120 kV, and digital images were acquired with a Veleta 2K × 2K charge-coupled device camera (Olympus-SIS). To characterize the CE ultrastructural differences of the agar-penetrating and nonpenetrating bacteria, approximately 10 to 20 cells were selected from TEM images at the same ×200,000 magnification, and the following parameters were measured using Fiji software (105): (i) total area of the longitudinal view of the cells, estimated in accordance with area = *ab* Π, where *a* and *b* denote the major and minor radiuses of the ellipsoidal cell, respectively (a > b) (106), (ii) thickness of the CE, determined by measuring the distance from the membrane to the edge of the cell wall, and (iii) width of the tripartite sections observed in E. faecalis’s CE, quantified by measuring the length (nm) of the electron-dark (CE-1 and CE-3) and electron-translucent (CE-2) zones.

### Fluorescence labeling and microscopy.

Bacterial samples from external and agar-penetrating E. faecalis grown on MOLP were collected as previously described (21) and suspended to an 0.8 OD_600_ in DPBS. To visualize putative *N-*acetylglucosamine-containing polymers and residues that constitute the peptidoglycan layer of the cell wall (29), samples were incubated with 5 μg/mL of the lectin wheat germ agglutinin (WGA) directly conjugated to Texas Red (Thermo Fisher Scientific) and analyzed as previously described in reference 21. The intracellular lipid content was evaluated with either the anionic lipid-specific FM 5-95 (*N*-3-trimethylammoniumpropyl-4-6-4-diethylaminophenyl-hexatrienyl pyridinium dibromide; Thermo Fisher Scientific) or the neutral lipid-specific BODIPY (boron-dipyrromethene 4,4-difluoro-1,3,5,7,8-pentamethyl-4-bora-3a,4a-diaza-s-indacene 493/503; Life Technologies) as reported (107, 108). Briefly, enterococcal cells were fixed with 4% paraformaldehyde-DPBS (BioWorld) for 15 min, pelleted down, and treated with either BODIPY (4 μg/mL) or FM 5-95 (16 μg/mL) in DPBS, followed by incubation for 60 min in the dark at room temperature or 37°C, respectively.

To assess the bacterial membrane sites of interaction with daptomycin (DAP), a ligand DAP-BODIPY labeling method was used with minor modifications (109). Briefly, DAP (2.5 mg/mL) was incubated with BODIPY-FL STP ester (1 mg/mL; B10006; Life Technologies) in 2 mL of sodium bicarbonate (0.2 M, pH 8.5) at 37°C for 1 h. Unbound BODIPY was then removed by 24 h of dialysis using a Micro Float-A-Lyzer G2, MWCO: 100 to 500 D (Spectrum Labs), against water at 4°C. Enterococcal cells were then treated with DAP-BODIPY (250 μg/mL) and CaCl_2_ (50 μg/mL) in DPBS for 30 min at 37°C in the dark (60). Then, cells were washed three times with DPBS and suspended on 4% paraformaldehyde-DPBS, followed by incubation with 2.0 μg/mL DAPI (4′,6-diamidino-2-phenylindole; Invitrogen) as described (21).

Imaging of all stained samples was performed using a Zeiss LSM880 confocal laser microscope with Airyscan. Fiji software (105) was used to determine the average pixel intensity of images at ×10 magnification, individually from three independent slides. Values were expressed as relative fluorescence intensity (RFI). The brightness and contrast of all images were adjusted linearly using Fiji.

### Flow cytometry.

Samples of nonpenetrating and agar-penetrating cells were diluted down until an absorbance of 0.8 OD_600_ in DPBS and stained with BODIPY at 4 μg/mL (108) or FM 4-64FX (*N*-3-triethylammoniumpropyl-4-p-diethylaminophenyl-hexatrienyl pyridinium dibromide; Thermo Fisher Scientific) at 20 μg/mL (60) for 60 min in the dark at room temperature and 37°C, respectively. Samples were washed twice with DPBS and subsequently fixed with 4% paraformaldehyde-DPBS (BioWorld) overnight at 4°C. Unstained cells were used as an intrinsic background control. All events were acquired on an LSR-Fortessa X-20 flow cytometer (BD Biosciences), and data were analyzed with FlowJo software (TreeStar).

### Lipid profiling by LC-MS.

Bacterial pools of either 20 colony-prints (agar-penetrating cells) or their corresponding externally grown bacteria were collected and suspended in DPBS to generate quadruplicates per condition (inside and outside). Cells from the colony-print were washed 3 times with DPBS and strained to remove the excess agar. Supernatants of all samples were then discarded, and pellets were stored at −80°C until further processing. Lipid extraction and LC-MS analyses were performed at the Weill Cornell Proteomics and Metabolomics Core Facility following standard protocols (110). Briefly, cell samples were sonicated using an ultrasonic disruptor (Qsonica) in 90% isopropanol. Stable isotope labeled lipid standards (Splash Lipidomix, Avanti Polar Lipids) were added to each sample as internal standards. Lipid profiling was performed using a Vanquish ultrahigh-performance liquid chromatography (UHPLC) system with a Cadenza CD-C18 3-μm packing column (Imtakt; 2.1 mm internal diameter [i.d.] by 150 mm) coupled to a Q Exactive Orbitrap mass spectrometer via an Ion Max ion source with a HESI II probe (Thermo Fisher Scientific). The mobile phase consisted of buffer A (60% acetonitrile, 40% water, 10 mM ammonium formate with 0.1% formic acid) and buffer B (90% isopropanol, 10% acetonitrile, 10 mM ammonium formate with 0.1% formic acid). The LC gradient was as follows: 0 to 1.5 min, 32% buffer B; 1.5 to 4 min, 32 to 45% buffer B; 4 to 5 min, 45 to 52% buffer B; 5 to 8 min, 52 to 58% buffer B; 8 to 11 min, 58 to 66% buffer B; 11 to 14 min, 66 to 70% buffer B; 14 to 18 min, 70 to 75% buffer B; 21 to 25 min, isocratic 97% buffer B, 25 to 25.1 min 97 to 32% buffer B; followed by 5 min of reequilibration of the column before the next run. The flow rate was 200 μL/min. A data-dependent mass spectrometric acquisition method was used, where the MS survey scan was followed by up to 10 MS/MS scans performed on the most abundant ions. Data were acquired in positive and negative mode in separate runs. The following electrospray parameters were used: spray voltage, 3.0 kV; heated capillary temperature, 350°C; HESI probe temperature, 350°C; sheath gas, 35 units; auxiliary gas, 10 units. For MS scans: resolution, 70,000 (at *m/z* 200); automatic gain control target, 3 × 10^6^; maximum injection time, 200 ms; scan range, 250 to 1,800 *m/z*. For MS/MS scans: resolution, 17,500 (at 200 *m/z*); automatic gain control target, 1 × 10^5^ ions; maximum injection time, 75 ms; isolation window, 1 *m/z*; normalized collision energy (NCE), stepped 20, 30, and 40.

MS/MS spectra were processed and exported as Mascot Generic Format (mgf) files using the MSConvert tool from ProteoWizard (version 3.0). The mgf files were then imported into the NIST MS Search Program and searched against the LipidBlast MS/MS library for lipid identification (111). Quantitation was performed using XCalibur (version 4.1) based on MS signal intensities. Lipid mass error tolerance was set to 5 ppm. The integrated intensity values were normalized by the total protein of each sample at the time of extraction quantified by means of the Pierce bicinchoninic acid (BCA) protein assay kit (Thermo Fisher Scientific) according to the manufacturer’s instructions. Normalized lipid intensities were used for principal-component analysis (PCA) with the R package DESeq2 and heatmap construction by determining the ratio of normalized, log-transformed intensities of agar-penetrating cells over the external bacterial samples’ values.

### RNA sequencing (RNA-seq) and transcriptomic analyses.

MOLP-penetrating and nonpenetrating cells were collected as described (21) from either sections of the colony-print (~1 cm^2^) or corresponding external colonies grown on filters (3.0 μm; Whatman) on top of the agarose medium (0.45% wt/vol agarose) 24, 48, and 72 h postinoculation, respectively. Total RNA isolation of triplicated pools of either 5 external colonies or colony-prints was performed using two approaches following prior reports (112–116). Briefly, samples from external cells were resuspended in DPBS, centrifuged, and then treated with RNAlater (Qiagen). Pellets were washed twice with ice-cold DPBS and treated with 300 μL of lysis buffer (10 mM Tris-HCl, pH 7.0, 33 μg/mL lysostaphin, 10 mg/mL lysozyme, and 1 mM EDTA) at 37°C for 60 min. Further lysis was accomplished by the addition of 300 μL GuCl buffer (6 M guanidine hydrochloride, 1 mM 2-betamercapto ethanol, 10 mM EDTA, and 0.1% [vol/vol] Tween 80) and 700 μL of Qiazol reagent (Qiagen) and then mechanical disruption for 5 min (45-s on and off cycles) with a Bio-Spec minibeadbeater (0.1 mm silica/zirconia). Proteins and genomic DNA were removed by standard treatment with chloroform (114–116), and the aqueous layer then recovered by centrifugation was used to precipitate the total RNA by means of an ethanol and ammonium acetate-based method previously described (113, 114).

The MOLP sections of the colony-print were recovered after washes with distilled water and 70% ethanol to remove the remaining cells on the surface. Sections were then placed on liquid nitrogen and quickly ground using a prechilled mortar and pestle, followed by homogenization with the lysis buffer (2% SDS, 16 mM EDTA, and 200 mM NaCl) at 100°C, followed by treatment with phenol-chloroform at 65°C for 10 min (112). The cell lysates were centrifuged and treated with chloroform/isoamyl alcohol (24:1). The RNA was recovered after isopropanol and ethanol 70% treatments as reported (112). Isolated RNA was then treated with a NucleoSpin gel and PCR cleanup kit (Macherey-Nagel) to eliminate any remaining agarose residues, following the manufacturer’s protocol.

Total RNA was treated with DNase I from the RNA Clean & Concentrator-5 kit (Zymo Research) following the manufacturer’s protocols. All samples were checked for RNA quality examined with an Agilent Bioanalyzer 2100, and mRNA libraries were generated and sequenced with Illumina Hi-Seq at the Weill Cornell Genomic Resources Core Facility. HISAT2 (117) was used to map reads (100 bp double end) to the genome of E. faecalis OG1RF (GenBank accession number NC_017316.1) by LC Sciences bioinformatic services. The mapped reads were assembled using StringTie (118) and merged using perl scripts and GffCompare. Differential gene expression was determined using StringTie by calculating the fragments per kilobase per million (FPKM). The differentially expressed mRNAs were selected with a log_2_ fold change of >1 or a log_2_ fold change of <–1, and statistical significance (*P* value, <0.05) was determined using the R package Ballgown (119). PCA and KEGG enrichment analyses of differentially expressed genes (inside versus outside samples with a *P* value of ≤0.05 and a fold change of ≥2.0) were performed using Partek Flow software (version 10.0); (https://www.partek.com/partek-flow/). An enrichment *P* value derived from a Fisher’s exact test was calculated. The enrichment score was determined as a negative natural logarithm of the enrichment *P* value. The higher the enrichment score, the more overrepresented the KEGG pathway is within the input list of differentially expressed genes (DEGs) (120).

### RNA extraction from enterococcal intestinal cell cocultures.

Bacteria were recovered 4 h postinfection from the apical and basolateral sides and the human intestinal epithelial cell monolayers. Briefly, translocation medium from apical and basolateral was spun down to pellet the cells, followed by treatment with 700 μL QIAzol lysis reagent (Qiagen) as indicated by the manufacturer. Nonadhered bacteria were removed by washing the monolayers with HBSS buffer (21), and the remaining attached enterococci were then lysed as described above. All samples were kept for 5 min at room temperature and then stored at −80°C until processing. Total RNA was recovered as previously reported (121).

### Real-time quantitative PCR (RT-qPCR).

cDNA was prepared from 250 ng of RNA using the qScript cDNA synthesis kit (Quantabio). RT-qPCR was performed using PerfeCTa SYBR green fastmix (Quantabio) on a QuantStudio 6 Flex real-time PCR system (Applied Biosystems). Normalized gene expression was calculated by the comparative threshold cycle method using *recA* (122) as the housekeeping gene. Primers used to amplify each target gene are listed in Table S3.

### Tolerance to envelope stressors.

Bacterial pools of either 20 external colonies in the surface or colony-prints inside the agar of MOLP medium were collected as described (21) and resuspended in DPBS. When indicated, enterococcal aggregates were disrupted by sonication as reported (21). Cell suspensions with an OD_600_ of 0.8 were diluted 1:2 in brain heart infusion (BHI) broth (supplemented with 50 μg/mL CaCl_2_) with and without 4 mg/mL diaminopimelic acid (DAP) (Selleck Chemicals), (60). Similarly, nonpenetrating and agar-penetrating enterococci were diluted down to an OD_600_ of 0.002 in DPBS and exposed to 0.6 μg/mL of the recombinant human β-defensins (hβDs), hβD2 or hβD3 (PeproTech) in BHI. All assays were done on 96-well plates and incubated at 37°C for 2 h. CFU numbers were estimated at 0 and 2 h postinoculation by serial dilutions and plating on BHI agar. The percentage of cell survival was calculated by determining the amount of antimicrobial-exposed enterococci relative to unexposed bacteria after 2 h of treatment for each condition.

### Statistical analyses.

All experiments were performed using at least three biological replicates and were repeated at least three independent times. Statistical analyses were performed using GraphPad Prism software (version 9.4.0 for MacOS).

### Data availability.

The data sets generated during the current study are available from the corresponding author upon reasonable request. The NCBI GEO (Gene Expression Omnibus) accession number for the RNA-seq data reported in this paper is GSE212145.
