# Supplementary figures and images for: Remodeling of the Enterococcal Cell Envelope during Surface Penetration Promotes Intrinsic Resistance to Stress
Source: mBio. 2022 Nov 10;13(6):e02294-22. doi: 10.1128/mbio.02294-22 (PMC9765498; doi:10.1128/mbio.02294-22)

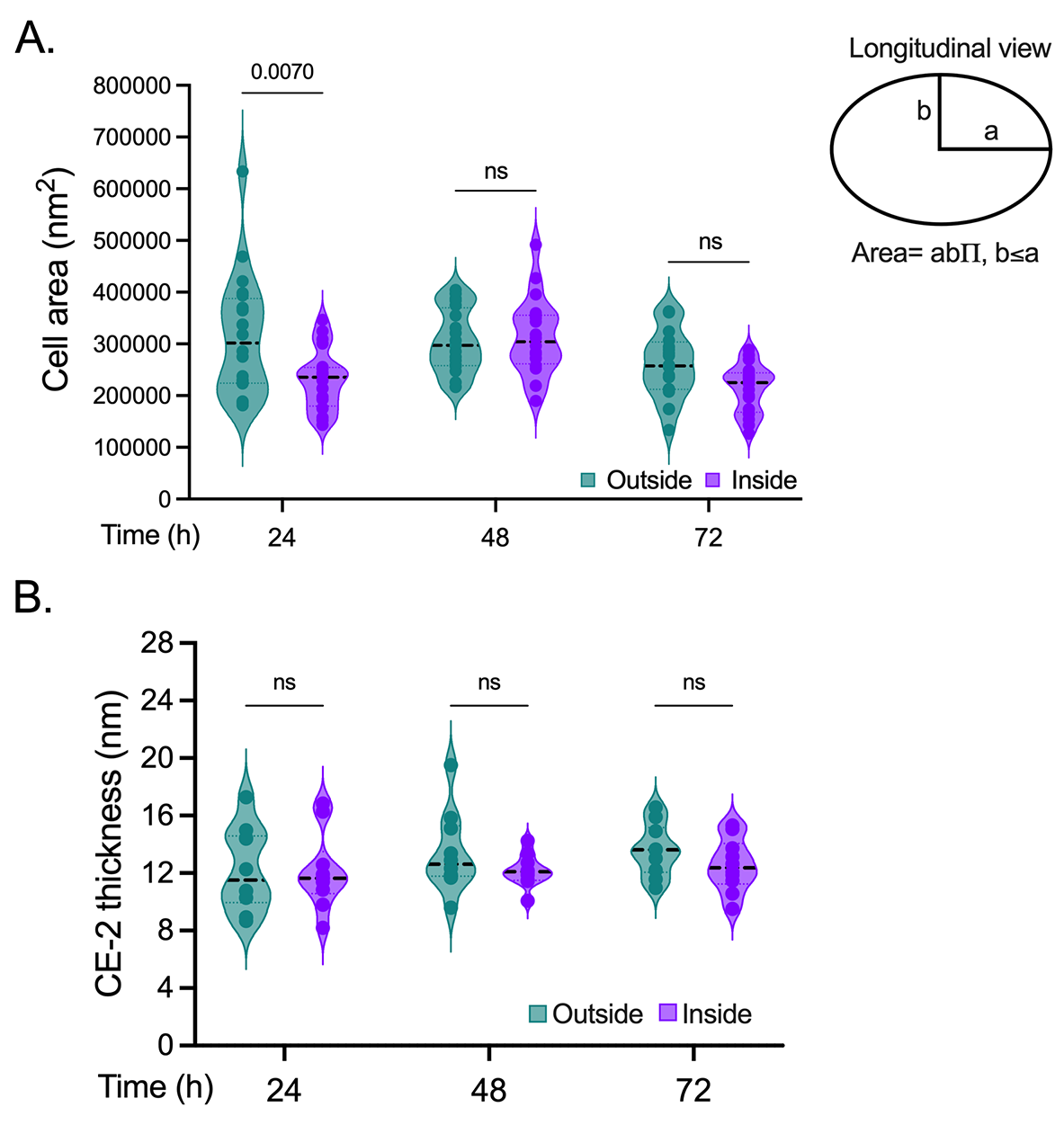

Supplement: FIG S1 [file mbio.02294-22-s0001.tif]

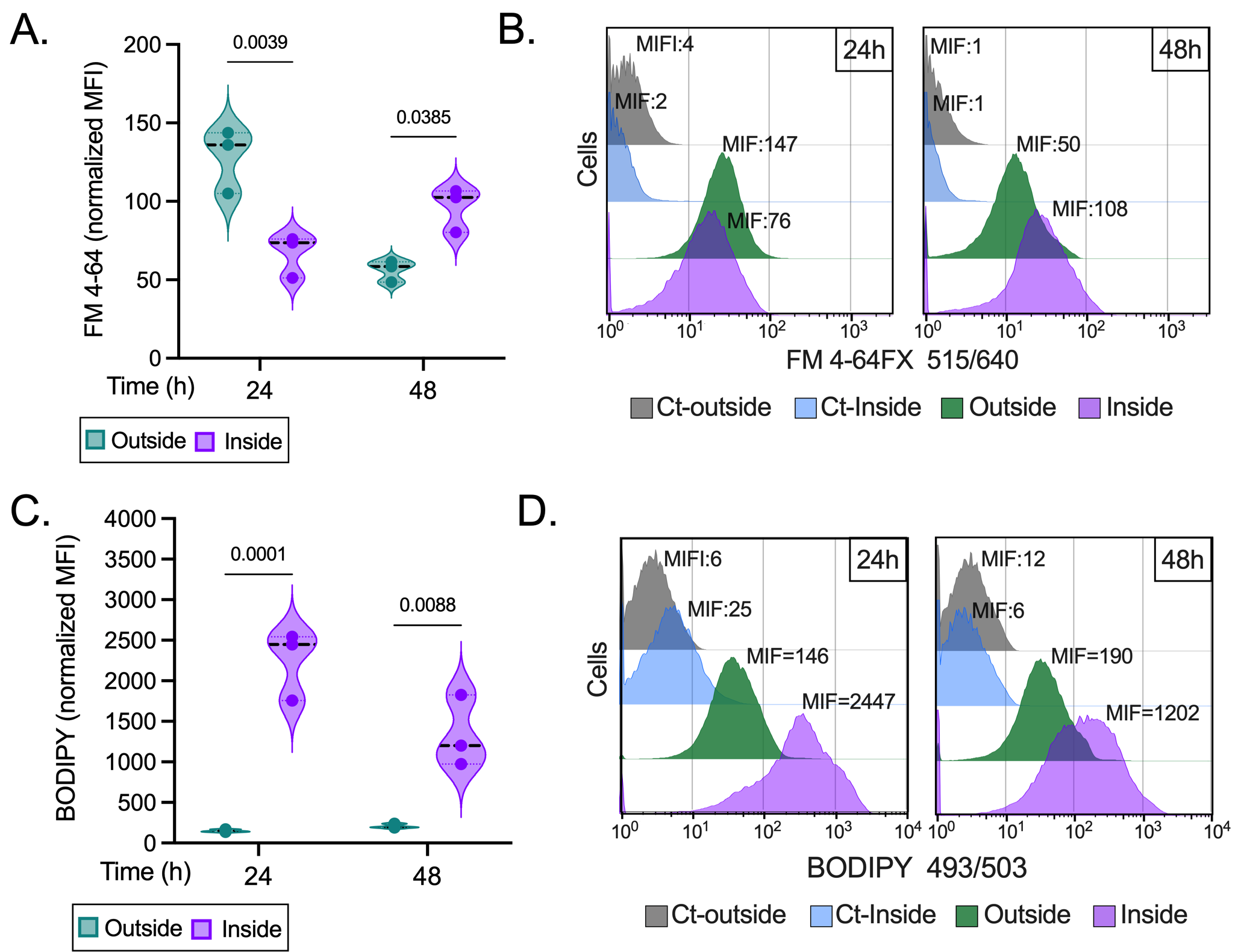

Supplement: FIG S2 [file mbio.02294-22-s0002.tif]

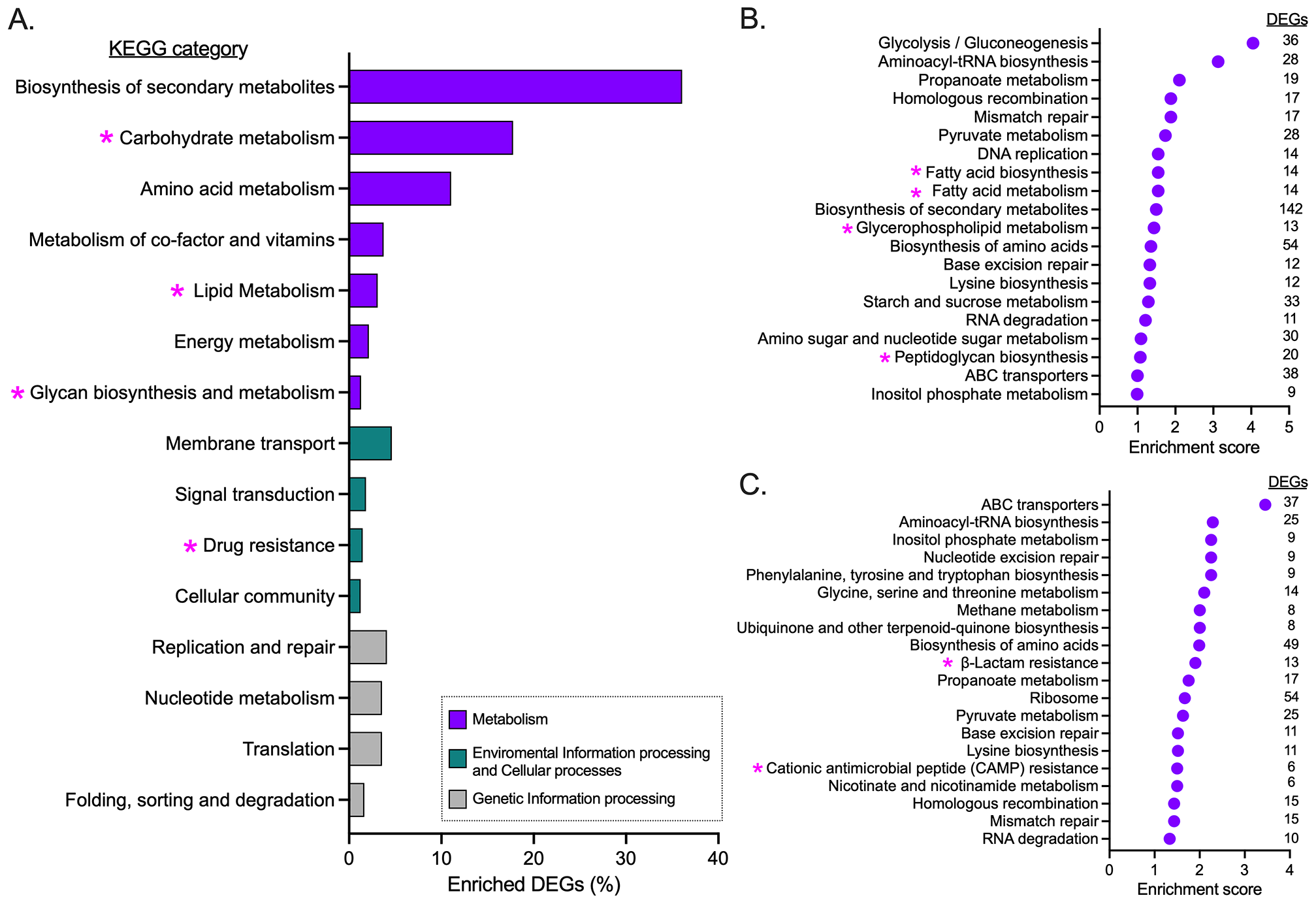

Supplement: FIG S3 [file mbio.02294-22-s0003.tif]

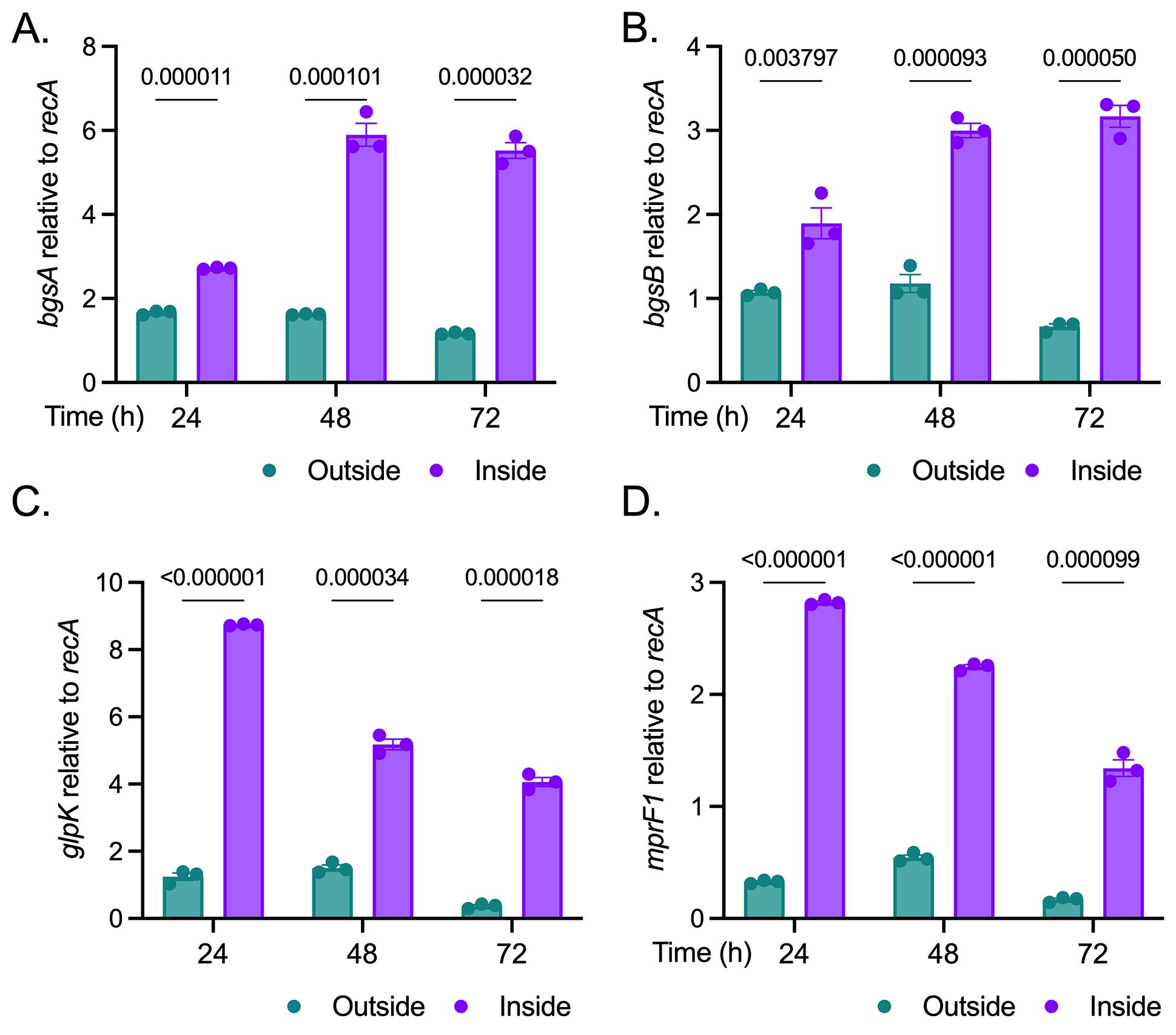

Supplement: FIG S4 [file mbio.02294-22-s0004.tif]

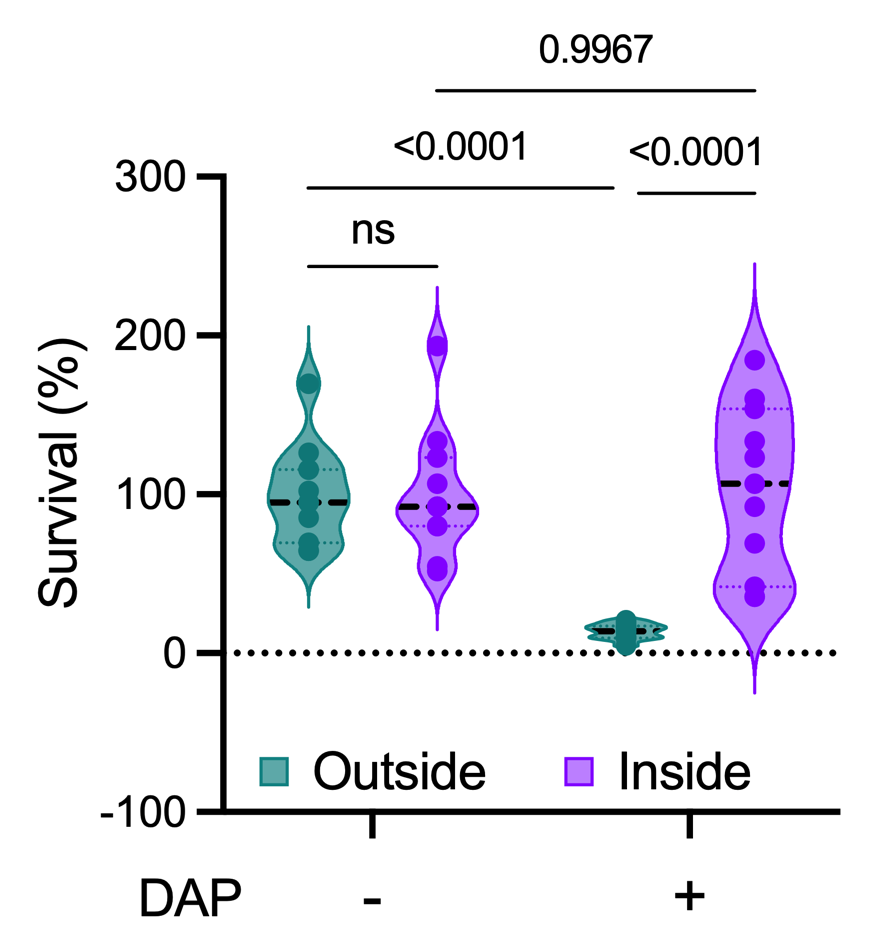

Supplement: FIG S5 [file mbio.02294-22-s0005.tif]

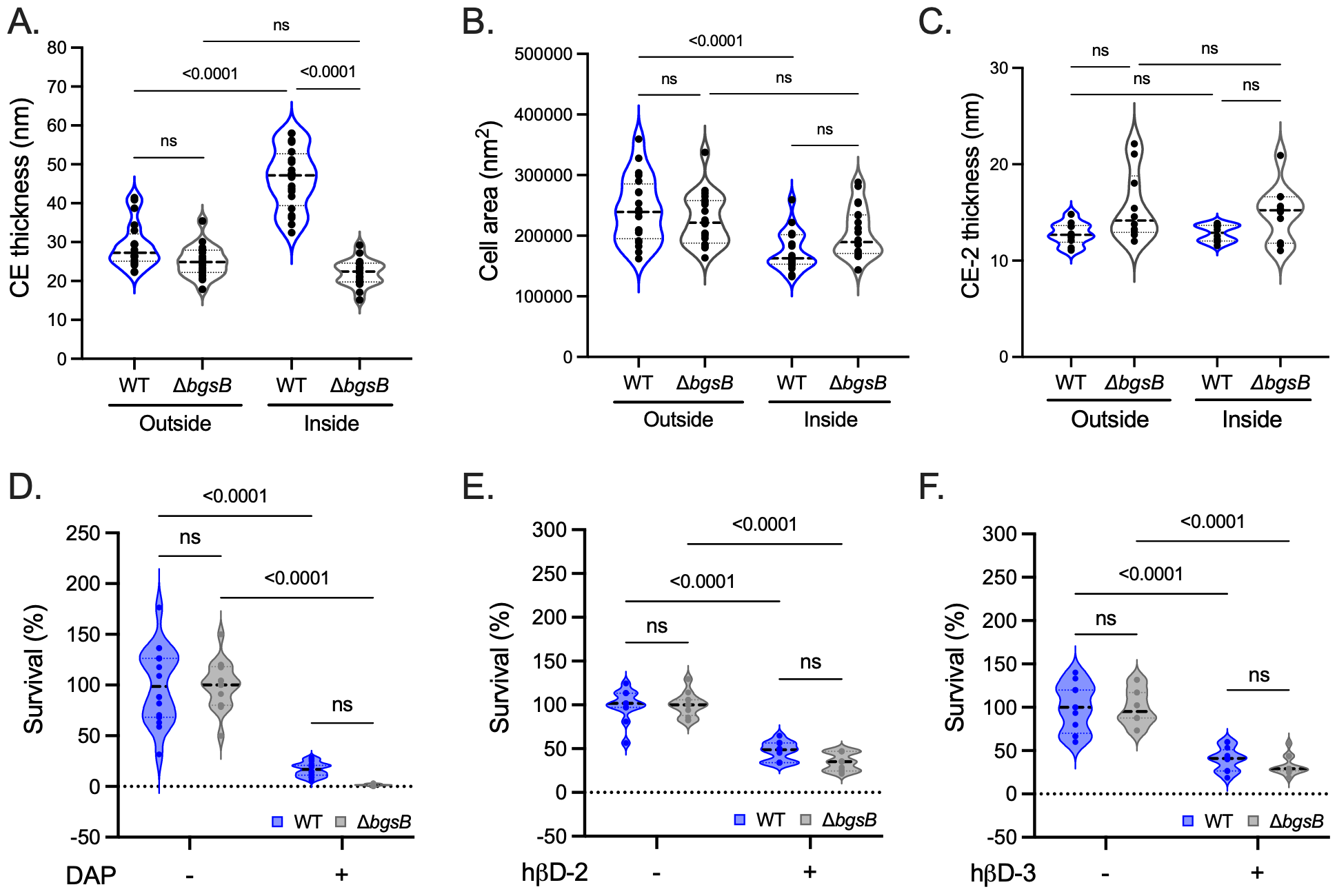

Supplement: FIG S6 [file mbio.02294-22-s0006.tif]

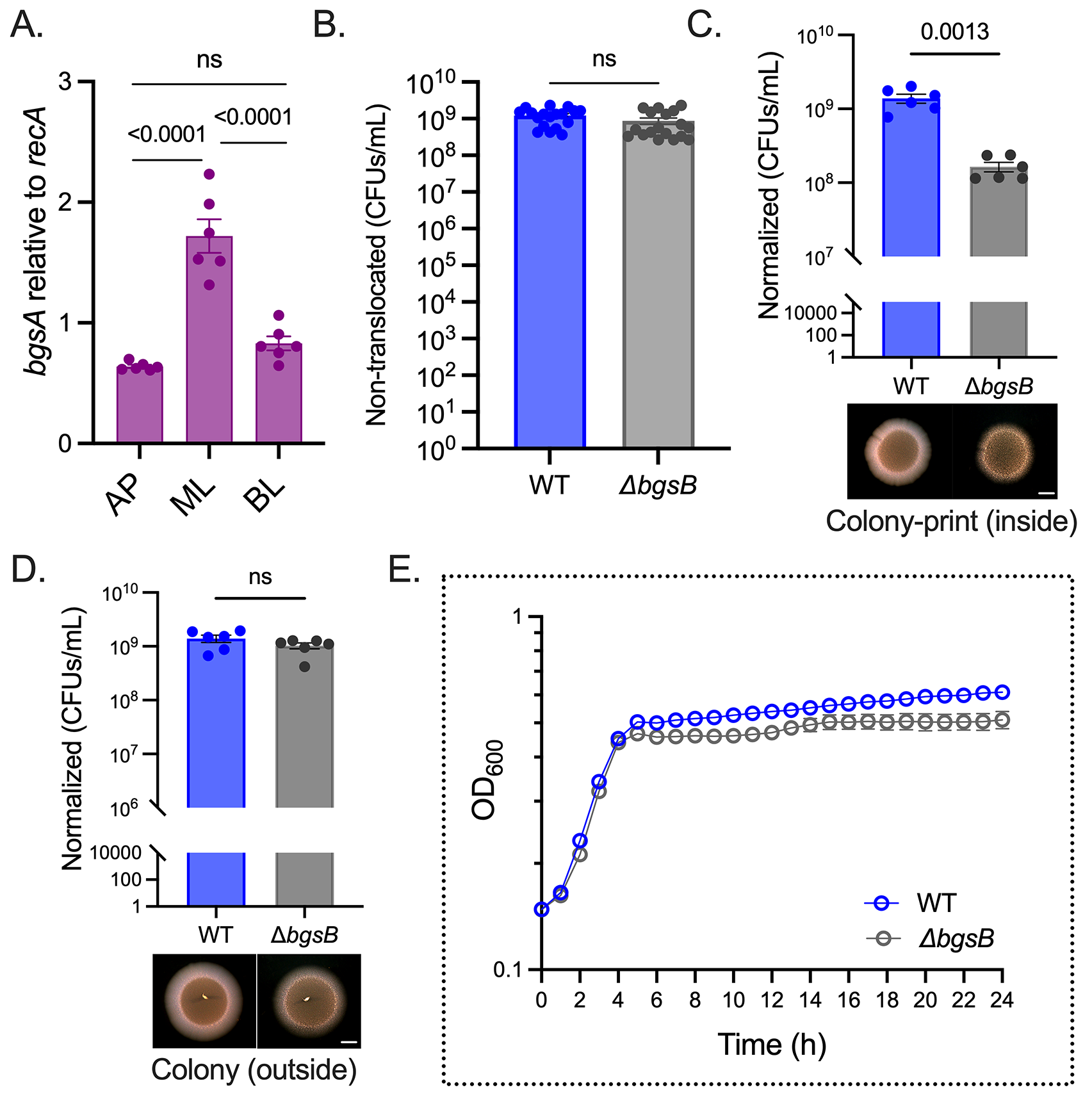

Supplement: FIG S7 [file mbio.02294-22-s0007.tif]
