# Supplementary material for: Remodeling of the Enterococcal Cell Envelope during Surface Penetration Promotes Intrinsic Resistance to Stress
Source: mBio. 2022 Nov 10;13(6):e02294-22. doi: 10.1128/mbio.02294-22 (PMC9765498; doi:10.1128/mbio.02294-22)
Supplement: TABLE S1 [file mbio.02294-22-s0008.pdf]

Lipid Class	Time (h)	Outside			Inside		
		24	48	72	24	48	72
TAG	24	---	ns	0.00389	---	ns	ns
	48	ns		0.0389	ns	---	ns
	72	0.0038	ns	---	ns	ns	---
DAG	24	---	0.012	ns	---	0.0003	<0.0001
	48	0.012	---	ns	0.0003	---	<0.0001
	72	ns	ns	---	<0.0001	<0.0001	---
DGDAG	24	---	<0.0001	<0.0001	---	<0.0001	<0.0001
	48	<0.0001	---	ns	<0.0001	---	<0.0001
	72	<0.0001	ns	---	<0.0001	<0.0001	---
PG	24	---	0.0191	<0.0001	---	ns	ns
	48	0.0191		<0.0001	ns	---	ns
	72	<0.0001	<0.0001	---	ns	ns	---
LPG	24	---	0.00003	0.0004	---	<0.0001	<0.0001
	48	0.0003	---	ns	<0.0001	---	0.0197
	72	0.0004	ns	---	<0.0001	0.0197	---
CL	24	---	ns	0.0003	---	ns	ns
	48	ns	---	0.0012	ns	---	ns
	72	0.0003	0.0012	---	ns	ns	---
