# Supplementary material for: Remodeling of the Enterococcal Cell Envelope during Surface Penetration Promotes Intrinsic Resistance to Stress
Source: mBio. 2022 Nov 10;13(6):e02294-22. doi: 10.1128/mbio.02294-22 (PMC9765498; doi:10.1128/mbio.02294-22)
Supplement: TABLE S2 [file mbio.02294-22-s0009.pdf]

Gene ID	Gene name	Log_2(FC) Inside vs outside			Individual replicates						q-value		
					24 h		48 h		72 h				
		24 h	48 h	72 h	Outside	Inside	Outside	Inside	Outside	Inside	24	48	72
OG1RF_RS08160	*glpO*	9.02	6.32	4.19							2E-51	7E-14	2E-29
OG1RF_RS8165	*glpK*	8.86	5.89	4.18							9E-45	8E-12	7E-24
OG1RF_RS8155	*glpF*	6.48	3.12	2.18							4E-14	ns	ns
OG1RF_RS03760	*murD*	4.38	3.09	1.96							2E-01	ns	ns
OG1RF_RS03765	*murG*	6.75	5.04	3.45							5E-15	2E-07	3E-08
OG1RF_RS07505	*gpsA*	1.85	3.34	0.86							3E-03	ns	4E-03
OG1RF_RS02235	*pbp2A*	6.63	4.66	3.16							8E-13	6E-07	6E-10
OG1RF_RS10785	*dltD*	5.16	4.21	2.64							6E-08	9E-04	3E-03
OG1RF_RS10790	*dltC*	6.32	5.04	3.20							6E-08	9E-04	3E-03
OG1RF_RS10795	*dtlB*	5.40	3.97	2.45							1E-05	2E-05	3E-01
OG1RF_RS10800	*dltA*	6.54	4.47	3.14							1E-05	2E-05	3E-01
OG1RF_RS08780	*tagD*	6.51	6.00	3.57							2E-07	3E-09	3E-06
OG1RF_RS11045	*penA*	6.38	5.76	4.23							2E-14	2E-19	3E-15
OG1RF_RS11065	*vanS*	6.18	4.18	2.90							1E-04	2E-03	2E-03
OG1RF_RS11070	*vanR*	5.10	4.93	1.40							1E-04	2E-03	2E-03
OG1RF_RS08785	---	6.15	6.03	3.14							2E-13	2E-16	9E-07
OG1RF_RS08835	*epaN*	6.08	5.57	3.81							1E-15	1E-20	6E-20
OG1RF_RS08830	*epaO*	5.94	4.70	2.89							3E-10	9E-08	2E-05
OG1RF_RS08880	*rmlA*	6.06	4.43	2.94							2E-11	4E-05	5E-05
OG1RF_RS08810	*epaS*	5.91	5.71	5.06							5E-03	9E-06	1E-06
OG1RF_RS04700	*murI*	5.80	4.35	3.77							2E-05	1E-02	2E-10
OG1RF_RS04935	*murA*	4.45	2.81	1.81							4E-02	5E-05	9E-05
OG1RF_RS08070	*murC*	2.28	1.74	0.48							3E-05	3E-08	3E-09
OG1RF_RS10725	*murB*	4.63	3.83	2.16							8E-04	2E-02	1E-01
OG1RF_RS02150	*murE*	3.46	2.27	1.39							2E-01	9E-04	3E-02
OG1RF_RS08680	---	5.74	4.96	3.33							6E-12	6E-10	5E-08
OG1RF_RS03750	*pbpC*	5.74	4.11	2.75							2E-06	2E-02	2E-04
OG1RF_RS04830	*pbp1A*	4.76	3.49	2.54							1E-04	3E-01	5E-04
OG1RF_RS07465	*pbp1B*	4.11	4.28	2.33							7E-02	5E-06	1E-02
OG1RF_RS08840	*epaM*	5.63	5.00	3.16							1E-15	1E-20	6E-20
OG1RF_RS08845	*epaL*	5.61	5.01	3.58							5E-10	5E-06	7E-09
OG1RF_RS07500	*galU*	5.57	4.89	3.11							1E-07	1E-09	1E-07
OG1RF_RS11890	*tag2*	5.50	3.66	2.43							5E-04	2E-01	ns
OG1RF_RS11215	*bgsB*	4.27	5.33	2.22							9E-02	3E-02	9E-02
OG1RF_RS11220	*bgsA*	5.65	5.77	3.40							3E-10	2E-17	3E-10
OG1RF_RS08775	---	5.42	4.83	2.70							7E-04	2E-07	2E-01
OG1RF_RS12310	*gldA2*	5.27	3.88	3.71							2E-06	1E-02	1E-11
OG1RF_RS07630	*fabG2*	5.24	2.65	2.16							1E-03	1E-01	5E-01
OG1RF_RS09755	---	5.21	5.27	3.30							3E-06	5E-18	1E-13
OG1RF_RS12555	---	5.11	3.45	2.54							7E-04	5E-01	7E-02
OG1RF_RS08055	*gdpD*	5.02	4.27	2.96							5E-05	6E-04	1E-05
OG1RF_RS08895	*epaB*	5.00	3.98	1.55							ns	2E-01	4E-01
OG1RF_RS02790	---	4.96	3.40	2.00							1E-02	6E-01	ns
OG1RF_RS08885	*epaD*	4.94	3.17	1.58							6E-03	ns	ns
OG1RF_RS08750	*glpQ*	4.95	4.08	2.71							9E-03	7E-02	3E-02
OG1RF_RS05370	*mdoB*	4.86	4.50	2.54							3E-05	1E-05	1E-03
OG1RF_RS10385	*fibA*	4.83	3.98	2.41							2E-03	2E-01	9E-02
OG1RF_RS08795	*epaOX*	4.77	5.07	2.54							4E-03	2E-08	1E-02
OG1RF_RS00530	---	4.69	2.55	2.08							1E-02	5E-02	5E-01
OG1RF_RS11345	*liaS*	4.60	3.98	2.65							6E-04	6E-02	4E-02
OG1RF_RS02700	*gdpD*	4.23	3.43	1.97							ns	ns	ns
OG1RF_RS03035	*alr*	4.50	2.77	3.20							2E-01	4E-01	6E-10
OG1RF_RS11575	---	4.49	3.80	2.39							3E-01	1E-01	2E-01
OG1RF_RS03030	*acpS*	4.49	3.77	2.88							3E-02	4E-02	1E-04
OG1RF_RS11165	*accB*	4.42	4.00	1.99							2E-02	9E-04	5E-01
OG1RF_RS02465	*yegS*	4.51	3.97	2.22							4E-03	3E-03	7E-02
OG1RF_RS08865	*rfbD*	4.37	3.46	1.72							1E-01	5E-01	8E-01
OG1RF_RS08820	*epaQ*	4.36	3.08	1.44							1E-02	1E+00	2E-01
OG1RF_RS01245	*tag*	4.35	2.71	1.38							1E-02	3E-01	3E-01
OG1RF_RS08815	*epaR*	4.34	3.30	1.96							2E-02	6E-01	5E-01
OG1RF_RS10535	*plsC*	4.36	2.92	2.39							ns	ns	ns
OG1RF_RS07510	*lgt*	4.24	2.50	1.14							3E-02	4E-02	7E-03
OG1RF_RS08790	*epaOX2*	4.15	4.48	1.76							1E-01	7E-06	6E-01
OG1RF_RS08770	*epaOY*	4.03	4.16	1.40							7E-04	2E-07	2E-01
OG1RF_RS11340	---	4.01	4.21	2.46							4E-02	6E-02	4E-02
OG1RF_RS03930	*mprF2*	3.86	4.11	1.97							ns	1E-04	ns
OG1RF_RS12680	*modB3*	3.89	3.44	1.80							4E-01	4E-01	9E-01

≥ 2
1.5
1.0
0.5
0.0
-0.5

OG1RF_RS06240	*pgi*	3.93	3.52	1.66	1E-11	2E-10	1E-05
OG1RF_RS01405	---	3.82	3.94	2.17	5E-02	3E-03	7E-02
OG1RF_RS01975	*cls*	3.70	3.08	1.06	ns	ns	5E-02
OG1RF_RS06840	*cls2*	2.69	2.10	0.67	6E-03	3E-06	9E-09
OG1RF_RS08890	*epaC*	3.79	3.02	1.53	7E-01	2E-01	4E-01
OG1RF_RS11170	*fabF2*	2.84	2.44	1.52	3E-02	6E-03	2E-01
OG1RF_RS11175	*fabG3*	3.79	3.58	2.02	8E-01	9E-02	3E-01
OG1RF_RS11180	*fabD*	1.00	1.61	0.93	2E-11	2E-07	1E-03
OG1RF_RS11185	*fabK*	3.10	3.78	2.17	3E-01	1E-02	7E-02
OG1RF_RS11160	*fabZ2*	3.25	2.69	1.65	6E-01	4E-01	6E-01
OG1RF_RS02060	*lplA*	3.73	4.41	2.65	ns	3E-03	2E-04
OG1RF_RS00290	---	3.57	2.98	1.75	ns	7E-01	6E-01
OG1RF_RS09570	*uppP*	3.57	2.70	1.28	ns	2E-01	5E-02
OG1RF_RS08800	---	3.57	3.43	2.01	ns	ns	ns
OG1RF_RS02010	*pgpA*	3.53	3.27	2.03	ns	ns	ns
OG1RF_RS10325	*glxK*	3.61	3.68	2.25	ns	ns	$5x10^{-02}$
OG1RF_RS08825	---	3.49	2.03	1.31	ns	2E-04	3E-02
OG1RF_RS08875	*rfbC*	3.42	3.31	2.13	ns	4E-01	1E-01
OG1RF_RS12155	*plsX*	3.35	3.25	2.11	ns	ns	ns
OG1RF_RS08870	*rmlB*	3.38	3.10	1.03	4E-01	6E-01	2E-04
OG1RF_RS08900	*mraY2*	3.24	2.85	1.19	5E-01	4E-01	2E-02
OG1RF_RS08860	---	3.21	2.65	1.03	3E-01	7E-02	8E-04
OG1RF_RS08765	---	3.19	3.64	0.53	5E-01	1E-01	4E-04
OG1RF_RS08760	*galE3*	3.08	2.47	1.15	1E-01	4E-03	3E-03
OG1RF_RS11585	---	3.06	3.04	1.51	5E-01	ns	2E-01
OG1RF_RS00150	*mprf1*	3.04	2.08	0.94	9E-02	1E-05	3E-05
OG1RF_RS11145	*accA*	2.65	1.96	1.33	2E-02	5E-06	1E-01
OG1RF_RS11150	*accD*	2.96	2.88	1.53	9E-02	3E-01	4E-01
OG1RF_RS11155	*accC*	1.92	1.84	1.37	3E-07	4E-07	1E-01
OG1RF_RS00280	*glmU*	2.89	2.43	1.50	3E-02	4E-03	2E-01
OG1RF_RS03550	*exp5*	2.87	2.03	0.00	ns	1E-02	8E-03
OG1RF_RS05955	*dhaK*	3.01	3.06	1.68	3E-25	2E-70	2E-71
OG1RF_RS09435	*dgkA*	2.78	2.61	1.20	8E-02	ns	3E-02
OG1RF_RS01265	*fabI*	2.73	2.52	0.50	1E-01	3E-01	5E-05
OG1RF_RS05950	*dhaK*	2.63	3.18	1.58	3E-29	2E-74	2E-75
OG1RF_RS05960	*dhak*	2.69	3.51	1.69	3E-28	2E-73	2E-74
OG1RF_RS03010	*ddl*	2.61	3.12	1.33	5E-04	5E-01	1E-02
OG1RF_RS07115	---	2.61	2.52	1.67	1E-02	9E-04	3E-01
OG1RF_RS08855	*epaJ*	2.54	2.22	0.21	3E-01	7E-02	2E-03
OG1RF_RS05945	*gldA*	2.51	3.06	2.24	5E-04	ns	6E-02
OG1RF_RS03015	*ppx*	2.48	2.44	1.44	8E-04	3E-02	2E-01
OG1RF_RS10985	*glk*	2.44	1.89	0.72	4E-03	3E-03	5E-09
OG1RF_RS11570	---	2.44	2.15	1.13	4E-01	4E-02	2E-02
OG1RF_RS06995	*plsY*	2.27	1.63	0.64	2E-03	2E-05	3E-04
OG1RF_RS00990	*aldA*	2.38	2.00	0.87	2E-04	7E-05	6E-05
OG1RF_RS07385	*lspA*	2.38	2.29	0.83	1E-01	ns	5E-01
OG1RF_RS03755	*mraY*	2.33	1.46	0.75	9E-05	8E-09	5E-07
OG1RF_RS04455	*galE2*	2.15	2.53	1.12	6E-04	1E-01	3E-02
OG1RF_RS12340	*pgsA*	2.19	2.04	0.93	2E-05	7E-05	3E-04
OG1RF_RS01270	*fabF*	2.07	2.24	0.90	2E-05	3E-04	2E-05
OG1RF_RS11195	*fabH*	2.06	2.64	1.71	3E-06	2E-01	6E-01
OG1RF_RS08705	*glmM*	1.96	2.94	1.05	7E-05	ns	8E-02
OG1RF_RS10960	*galE4*	1.89	1.94	1.27	1E-05	7E-05	1E-01
OG1RF_RS06935	*eutC*	2.00	3.22	1.35	3E-33	2E-78	2E-79
OG1RF_RS06940	*eutB*	2.00	3.22	2.01	3E-32	2E-77	2E-78
OG1RF_RS06945	*eutA*	1.96	3.81	0.96	3E-34	2E-79	2E-80
OG1RF_RS01275	*fabZ*	1.80	1.89	0.66	2E-07	7E-05	2E-05
OG1RF_RS09790	*cdsA*	1.65	1.98	1.45	9E-07	6E-03	ns
OG1RF_RS08685	*glmS*	1.49	1.69	-0.43	7E-14	7E-10	6E-28
OG1RF_RS11580	---	1.42	1.32	0.29	4E-03	3E-04	1E-04
OG1RF_RS00345	*fabG*	1.09	1.34	1.09	8E-12	1E-11	6E-04
OG1RF_RS07810	*mdoB2*	2.84	3.03	1.09	ns	ns	ns
