# Supplementary material for: Remodeling of the Enterococcal Cell Envelope during Surface Penetration Promotes Intrinsic Resistance to Stress
Source: mBio. 2022 Nov 10;13(6):e02294-22. doi: 10.1128/mbio.02294-22 (PMC9765498; doi:10.1128/mbio.02294-22)
Supplement: TABLE S3 [file mbio.02294-22-s0010.pdf]

Strain Name	Parental background	Source	Description	References
Enterococcus faecalis				
DK10	OG1RF	Oral	WT, Rif^r, Fus^r	102
DK431	OG1RF Δ *bgsB*	---	*bgsB* deletion mutant	65

Primer name	Primer sequence 5'-3'	
recA-f	ACGTGGCCGAATTATTGAAG	This Work
recA-r	GATGTCAATCGCACCACTTG	This Work
bgsA-f	CGGCGATCTCAAATGGTATT	This Work
bgsA-r	CTGTCGCGCAACTTCAATAA	This Work
bgsB-f	TTTGGTGCGGGTATTTTAGG	This Work
bgsB-r	AACATGTGATGGACGGACAA	This Work
glpK-f	ATGAACACTGGCGAAGAACC	This Work
glpK-r	TGTTGACGCTTTGGCTACTG	This Work
mprF1-f	TCCTTTGGTAACCTCGATGC	This Work
mprF1-r	CCTGGAATCATCGAAGCAAT	This Work
